# Characterization of virus-mediated immunogenic cancer cell death and the consequences for oncolytic virus-based immunotherapy of cancer

**DOI:** 10.1038/s41419-020-2236-3

**Published:** 2020-01-22

**Authors:** Jing Ma, Mohanraj Ramachandran, Chuan Jin, Clara Quijano-Rubio, Miika Martikainen, Di Yu, Magnus Essand

**Affiliations:** 1 Department of Immunology, Genetics and Pathology, Science for Life Laboratory, Uppsala University, 75185 Uppsala, Sweden; 2 Present Address: Laboratory of Molecular Neuro-Oncology, Department of Neurology, University Hospital and University of Zurich, 8091 Zurich, Switzerland

**Keywords:** Cancer immunotherapy, Immunotherapy, Translational research

## Abstract

Oncolytic viruses have the potential to induce immunogenic cell death (ICD) that may provoke potent and long-lasting anti-cancer immunity. Here we aimed to characterize the ICD-inducing ability of wild-type Adenovirus (Ad), Semliki Forest virus (SFV) and Vaccinia virus (VV). We did so by investigating the cell death and immune-activating properties of virus-killed tumor cells. Ad-infection of tumor cells primarily activates autophagy, but also activate events of necroptotic and pyroptotic cell death. SFV infection on the other hand primarily activates immunogenic apoptosis while VV activates necroptosis. All viruses mediated lysis of tumor cells leading to the release of danger-associated molecular patterns, triggering of phagocytosis and maturation of dendritic cells (DCs). However, only SFV-infected tumor cells triggered significant T helper type 1 (Th1)-cytokine release by DCs and induced antigen-specific T-cell activation. Our results elucidate cell death processes activated upon Ad, SFV, and VV infection and their potential to induce T cell-mediated anti-tumor immune responses. This knowledge provides important insight for the choice and design of therapeutically successful virus-based immunotherapies.

## Introduction

Many human viruses are being evaluated for their abilities to selectively infect, replicate in and kill cancer cells and therefore be used as therapeutic oncolytic viruses (OVs) for the treatment of various human malignancies. Upon infection of a cell, viruses possess specific abilities to interact with cellular proteins to avoid early host cell death and immune system recognition in order to promote their replication and release progeny virus, and eventually killing the host cell. Cell death can be classified according to morphologic and structural changes occurring in dying cells^1^ and viruses typically activates one or more cell death pathways during infection, replication or cell lysis^2^. Some forms of programmed cell death lead to silent and organized uptake of dead cells by phagocytic cells and they are considered as intrinsically tolerogenic^3^. Other forms of cell death can induce an immune response through activation of dendritic cells (DCs) and adaptive immune cells and are termed “immunogenic cell death” (ICD)^4^. Inducers of ICD are characterized by their ability to stimulate the release of damage-associated molecular patterns (DAMPs) from dying host cells, such as extracellular ATP (“find-me” signal), cell surface exposure of Calreticulin (CRT) (“eat-me” signal to antigen-presenting cells), and release of high mobility group box 1 protein (HMGB1) (activation signal for immune cells). Collectively they serve as strong immune stimulants and ICD is regarded as a keystone of anti-tumor immunity^5,6^.

The ability of OVs to induce ICD can greatly affect their potential to evoke an adaptive anti-tumor immune response, which is known to significantly contribute to the anti-tumor effect of OVs^7,8^. Viruses usually exacerbate the immunogenicity of dying cancer cells in order to promote their own replication and survival^9^. On the other hand, virus replication and lysis leads to release of pathogen-associated molecular patterns (PAMPs) such as viral proteins and nucleic acids^10^, together with DAMPs, which further intensify immune responses. Viral infection can induce endoplasmic reticulum (ER) stress, release of reactive oxygen species (ROS), activation of type-I interferons (IFN-I) and a plethora of inflammatory cytokines and chemokines^11^. These factors can attract immune cells such as DCs to the tumor tissue, promote phagocytosis of tumor cell debris, including tumor-specific antigens, and activate DCs for subsequent migration to draining lymph node and priming of anti-cancer T-cell responses^12^. Cellular products processed and released prior to virus-induced lysis of tumor cells determine the context in which the immune system recognizes the appropriate PAMPs and DAMPs^13^.

In this study, we asked which cell death pathways are triggered during wild-type Adenovirus (Ad) serotype-5, Semliki Forest virus (SFV) strain SFV4 and Vaccinia virus (VV) Western Reserve strain infection and the ability of these viruses to induce tumor cell lysis and subsequent immune activation. The understanding of wild-type viruses is critical for optimal development of OV-based cancer immunotherapies. Adenovirus is a non-enveloped virus, containing a linear double-stranded DNA genome and Adenoviruses are some of the most commonly used OVs in pre-clinical and clinical research^14^. SFV4 is a neurotropic enveloped virus, with a positive-sense single-stranded RNA genome^15^. SFVs have been engineered as OV for treatment of glioblastoma^15,16^ but is yet to be evaluated in clinical trials. VV is an enveloped virus, containing a large linear, double-stranded DNA genome. Thymidine kinase (TK) and/or vaccinia virus growth factor (VGF) gene-deleted VVs have been used as OV in research and clinics^17^. Although all wild-type viruses induce lysis of tumor cells, we found that their potential to trigger DC activation and T-cell immune responses are quite different. SFV4 was the most potent inducer of adaptive immunity followed by Adenovirus. While, VV was quite efficient in blocking immune responses.

## Results

### Adenovirus serotype 5 initiates multiple cell death pathways including inflammasome activation

We investigated which of the most common cell death pathways (illustrated in Supplementary Fig. 1) were activated upon infection of HOS and A549 tumor cells by wild-type Adenovirus (Ad) serotype 5, Semliki Forest virus (SFV) strain SFV4 and Vaccinia virus (VV) Western Reserve strain.

**Fig. 1:**
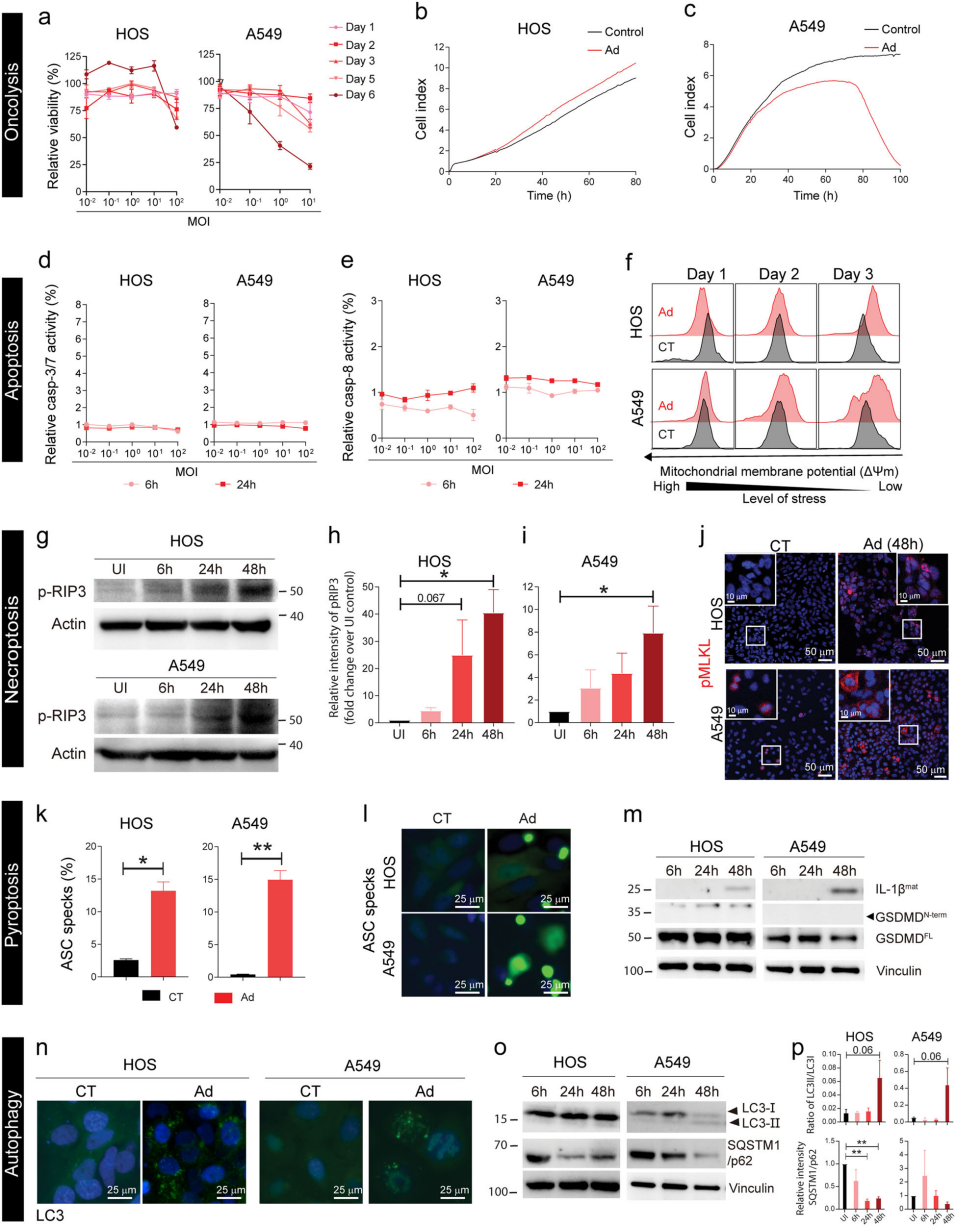
Ad-induced cell death in HOS and A549 cells. *Oncolysis:* (**a**) Cell viability of Ad-infected cells (MOI 10^-2^–10^2^) at days 1, 2, 3, 5, and 6 was measured using AlamarBlue™ viability assay. Cell viability is represented as percentage relative to non-infected control cells. Data are presented as mean ± SEM (*n* = 3). (**b)** HOS cells and (**c**) A549 cells were infected with Ad at MOI 10. Cell index values as a measure of cell viability were measured by the xCELLigence system. *Apoptosis:* Analysis of (**d**) Caspase-3/7 and (**e**) Caspase-8 in Ad-infected (MOI 10^-2^–10^2^) HOS and A549 cells at 6 h and 24 h was performed using Caspase-3/7ApoTox-Glo™ Triplex and Caspase-Glo® 8 assays. Caspase activity is represented as percentage relative to non-infected control cells. Data are presented as mean ± SEM (*n* = 3). (**f)** Mitochondrial membrane potential was measured by flow cytometry after infection with Ad (MOI = 10) at days 1, 2 and 3. *Necroptosis****:*** (**g**) Phosphorylated RIP3 (p-RIP3) was detected in Ad-infected (MOI 10) HOS and A549 cells by Western blot 6, 24 and 48 h after infection. Densitometric analysis of fold change in p-RIP3 post Ad-infection in (**h**) HOS and (**i**) A549 compared to un-infected control (*n* = 3). (**j)** HOS and A549 cells cultured on glass slides were infected with Ad (MOI = 10) for 48 h and phosphorylated MLKL (p-MLKL) was detected by antibody staining (red). Cell nuclei were stained with Hoechst 33342 (blue). The multicolor fluorescent analyses were carried out in three individual experiments. Representative images from one experiment is shown. *Pyroptosis****:*** HOS and A549 cells expressing GFP-ASC were infected with the Ad (MOI 10) for 48 h. ASC specks were quantified by flow cytometry (**k**) (*t*-test, *p < 0.05, **p < 0.01, *n* = 3) and representative images (**l**) were taken from cells cultured on glass chamber slides. (**m)** Lysates were harvested from Ad-infected HOS and A549 cells (MOI = 50) at 6, 24 and 48 h. Full-length GSDMD^FL^, truncated and active GSDMD^N-term^ (lower band, indicated by arrow) and mature IL-1β^mat^ were detected by Western blot. Vinculin was used as internal loading control. *Autophagy****:*** HOS and A549 cells expressing eGFP-LC3 cells were infected with Ad (MOI = 10) and monitored by fluorescence microscopy. (**n)** Images were acquired at 48 h. (**o)** The cell lysates of Ad-infected HOS and A549 cells (MOI = 50) were harvested at 6, 24, and 48 h for analysis of the non-lipidated form of LC3 (LC3-I), lipidated form (LC3-II) and the cargo-loading adaptor protein SQSTM1/p62. The housekeeping membrane-cytoskeletal protein vinculin was used as gel loading control. (**p)** Densitometric analysis of ratio of LC3-II/LC3-I and SQSTM1/p62 degradation Ad-infection in HOS and A549 compared to un-infected control. Statistical analysis for normally distributed data were performed by means of one-way ANOVA with Dunnet’s post hoc test and for not normally distributed data were performed by means of Kruskal–Wallis test with Dunn’s post hoc test (*p < 0.05, **p < 0.01 and *n* = 3).

*Oncolysis:* Ad had no cytotoxic effect in HOS cells even at a high multiplicity of infection (MOI) of 100 virus particles per cell (Fig. 1a), while A549 cells were efficiently killed by Ad at day 6 post-infection (p.i.) also at low MOIs (Fig. 1a). This was confirmed by xCELLigence real time cell viability assay (Fig. 1b, c). The difference in result for the two cell lines could be partially explained by the fact that HOS was less permissive to Ad-infection than A549 as observed by green fluorescent protein (GFP) expression after transduction with an Ad5(GFP) vector (Supplementary Fig. 2a, b). *Apoptosis:* Ad-infection did not increase caspase-3/7 or caspase-8 activities either in A549 or HOS cells (Fig. 1d, e) but led to a decrease in mitocondrial membrane potential (Δψm) in A549 after 72 h of infection (Fig. 1f). These results indicate that apoptotic pathways are not activated upon Ad-infection. *Necroptosis:* Initiation of necroptosis was analyzed by measuring phosphorylated receptor-interacting protein kinase 3 (p-RIP3). Uninfected HOS and A549 cells had very low levels of p-RIP3 but levels increased overtime after Ad-infection (Fig. 1g–i, Supplementary Fig. 3a, b). This was followed by increase in phosphorylation status of mixed-lineage kinase domain-like (MLKL) (Fig. 1j). Together, this suggests that necroptosis is activated upon Ad-infection. *Pyroptosis*: GFP-tagged inflammasome adaptor protein apoptosis-associated speck-like protein containing a caspase recruitment domain (ASC) cells (A549-GFP-ASC or HOS-GFP-ASC) were infected and inflammasome formation was measured as aggregation of ASC. Quantification of ASC specks by flow cytometry (Fig. 1k, Supplementary Fig. 4a) and by imaging (Fig. 1l, Supplementary Fig. 4b, c) show increased formation of inflammasome upon Ad-infection both in HOS and A549 cells. Similar results were also observed in wild-type A549 and HOS cells when stained for ASC using antibody (Supplementary Fig. 4f). This was followed by cleavage of pro-interleukin-1β (IL-1β) to mature IL-1β^mat^ (17-kDa) (Fig. 1m, Supplementary Fig. 3c, d at 48 h p.i.). The cleaved and biologically active N-terminal portion of gasdermin D (GSDMD) (GSDMD^N-term^) was not detected (indicated by arrow) either in HOS or A549 cells (Fig. 1m, Supplementary Fig. 3e, f). Ad-infection induces inflammasome formation and mature IL-1β release, but pyroptopic cell death is most likely not executed. *Autophagy* was checked using cells with GFP-tagged microtubule-associated protein 1A/1B light chain 3 (LC3) to monitor autophagosome formation. Ad infection induced bright puncta structures in the cytoplasm of both HOS and A549, indicative of LC3 accumulation and autophagosome formation (Fig. 1n). Conversion of LC3-I to LC3-II was observed 48 h p.i. in Ad-infected HOS and A549 cells (Fig. 1o, p, Supplementary Fig. 3g, h). The autophagic cargo adapter sequestosome-1 (SQSTM1)/p62 directly interacts with LC3 and is degraded after fusion of autophagosomes with lysosomes. Thus, measurement of total cellular levels of SQSTM1/p62 negatively correlates with autophagic flux. SQSTM1/p62 levels decreased overtime in Ad-infected HOS and A549 cells (Fig. 1o, p, Supplementary Fig. 3i, j). Vacuolization of the cytoplasm, a hallmark of autophagy induction was also observed after Ad-infection by electron microscopy (Supplementary Fig. 5a–c). The results suggest that Ad-infection initiates autophagy in both cell lines. In conclusion, adenovirus initiates multiple cell death pathways including necreoptosis, inflammasome activation and autophagy before the tumor cells die by Ad-mediated lysis.

### Semliki Forest virus induces rapid cell lysis accompanied by induction of apoptosis

**Fig. 2:**
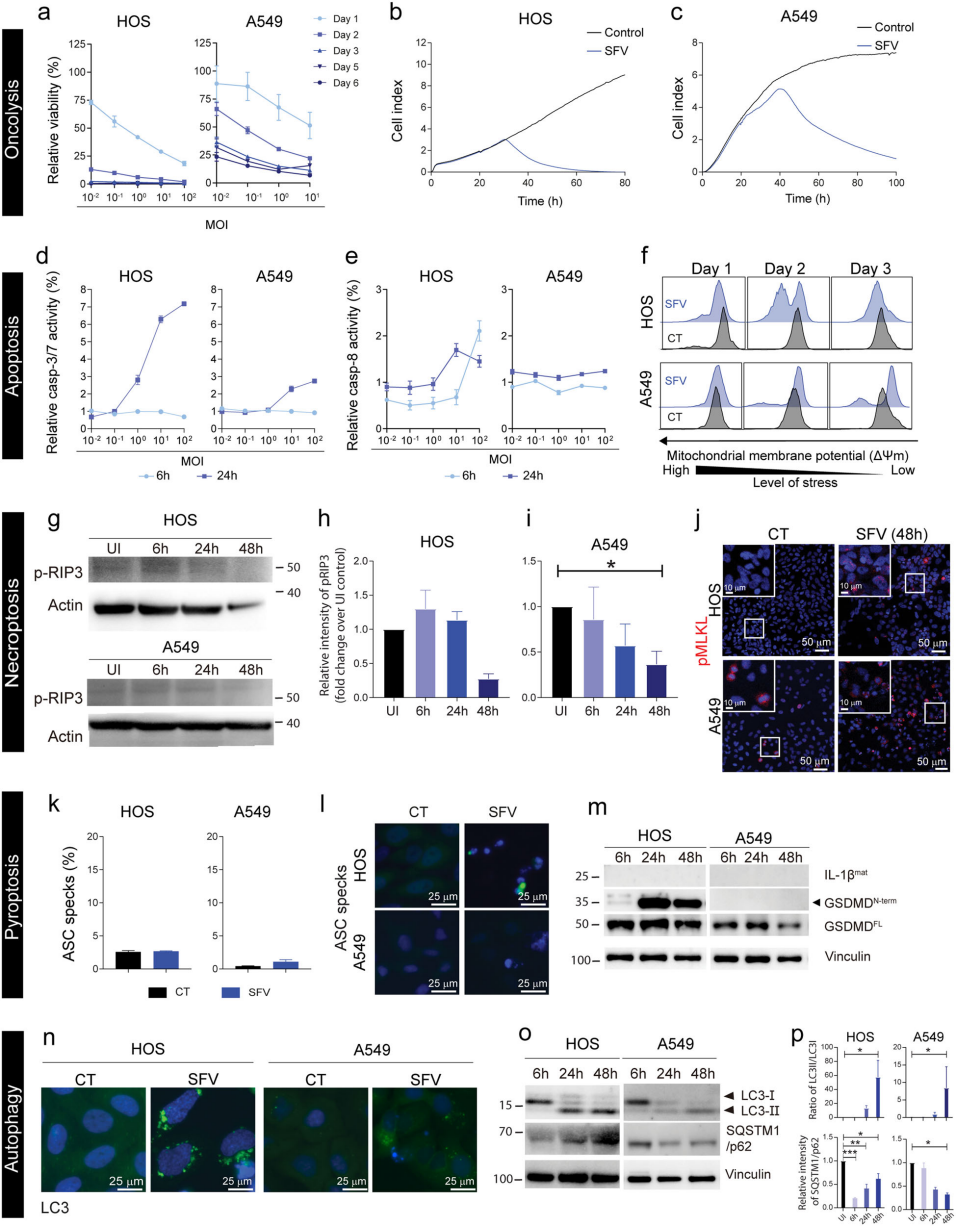
SFV4-induced cell death in HOS and A549 cells. *Oncolysis:* (**a**) Cell viability of SFV4-infected cells (MOI 10^-2^–10^2^) at days 1, 2, 3, 5, and 6 was measured using AlamarBlue™ viability assay. Cell viability is represented as percentage relative to non-infected control cells. Data are expressed as mean ± SEM (*n* = 3). (**b**) HOS cells and (**c**) A549 cells were infected with SFV 4at MOI 10. Cell index values as a measure of cell viability were measured by the xCELLigence system. *Apoptosis:* Analysis of (**d**) Caspase-3/7 and (**e**) Caspase-8 in SFV4-infected (MOI 10^-2^-10^2^) HOS and A549 cells at 6 h and 24 h was performed using Caspase-3/7ApoTox-Glo™ Triplex and Caspase-Glo® 8 assays. Caspase activity is represented as percentage relative to non-infected control cells. Data are presented as mean ± SEM (*n* = 3). (**f**) Mitochondrial membrane potential was measured by flow cytometry after infection with SFV4 (MOI = 10) at days 1, 2, and 3. *Necroptosis:* (**g**) Phosphorylated RIP3 (p-RIP3) was detected in SFV4-infected (MOI = 10) HOS and A549 cells by Western blot 6, 24, and 48 h after infection. Densitometric analysis of fold change in p-RIP3 post SFV4 infection in (**h**) HOS and (**i**) A549 compared to un-infected control (*n* = 3). (**j**) HOS and A549 cells cultured on glass slides were infected with SFV4 (MOI = 10) for 48 h and phosphorylated MLKL (p-MLKL) was detected by antibody staining (red). Cell nuclei were stained with Hoechst 33342 (blue). The multicolor fluorescent analyses were carried out in three individual experiments. Representative images from one experiment are shown. *Pyroptosis****:*** HOS and A549 cells expressing GFP-ASC were infected with the SFV4 (MOI 10) for 48 h. ASC specks were detected by flow cytometry (**k**) and representative images (**l**) were taken from cells cultured on glass chamber slides. (**m**) Lysates were harvested from SFV4-infected HOS and A549 cells (MOI = 10) at 6, 24 and 48 h. Full-length GSDMD^FL^, truncated and active GSDMD^N-term^ (lower band, indicated by arrow) and mature IL-1β^mat^ were detected by Western blot. Vinculin was used as internal loading control. *Autophagy:* HOS and A549 cells expressing eGFP-LC3 cells were infected with SFV4 (MOI = 10) and monitored by fluorescence microscopy. (**n**) Images were acquired at 48 h. (**o**) The cell lysates of SFV4-infected HOS and A549 cells (MOI = 50) were harvested at 6, 24, and 48 h for analysis of the non-lipidated form of LC3 (LC3-I), lipidated form (LC3-II) and the cargo-loading adaptor protein SQSTM1/p62. The housekeeping membrane-cytoskeletal protein vinculin was used as gel loading control. (**p**) Densitometric analysis of ratio of LC3-II/LC3-I and SQSTM1/p62 degradation SFV4 infection in HOS and A549 compared to un-infected control. Statistical analysis for normally distributed data were performed by means of one-way ANOVA with Dunnet’s post hoc test and for not normally distributed data were performed by means of Kruskal–Wallis test with Dunn’s post hoc test (*p < 0.05, **p < 0.01 and *n* = 3).

*Oncolysis:* SFV4 displayed rapid cytotoxic effects in HOS cells even at a very low MOI of 0.01 virus particles per cell (Fig. 2a). A549 cells were also susceptible to SFV4 oncolysis in a dose and time-dependent manner (Fig. 2a). The results were confirmed by xCELLigence measurements (Fig. 2b, c). *Apoptosis:* Effector caspase-3/7 activity increased markedly 24 h post-SFV4 infection in a dose-dependent manner in both cell lines (Fig. 2d). However, initiator caspase-8 activity increased only in HOS cells after infection and only at higher virus load (>10 virus particles per cell) (Fig. 2e). In addition, there was a decrease in the mitocondrial membrane potential Δψm in HOS cells 2 days p.i. and in a fraction of A549 cells 3 days p.i. (Fig. 2f). This indicates activation of an intrincic caspase-3/7-mediated apoptotic pathway especially in HOS and to some extent also in A549 upon SFV4 infection. *Necroptosis:* Uninfected HOS and A549 cells had very low phosphorylation of RIP3 and if anything there was a decrease in p-RIP3 overtime in HOS and A549 cells upon SFV4 infection (Fig. 2g–i, Supplementary Fig. 3a, b) and no increase in p-MLKL after SFV4 infection in either HOS or A549 compared to their control (Fig. 2j). Therefore, necroptosis is not activated upon SFV4 infection. *Pyroptosis:* ASC specks were sparsely detected in SFV4-infected A549-GFP-ASC or HOS-GFP-ASC cells and the relative increase quantified by flow cytometry compared to control cells was marginal (Fig. 2k, l, Supplementary Fig. 4b, d). Mature IL-1β^mat^ (17-kDa) was absent in both SFV4-infected HOS and A549 cells (Fig. 2m, Supplementary Fig. 3c, d). However, cleaved GSDMD^N-term^ was detected in SFV4-infected HOS but not in A549 (Fig. 2m, Supplementary Fig. 3e, f). These results suggest that inflammasomes are not formed and pyroptosis is not associated with SFV4 infection. *Autophagy:* Oligomerization of LC3 was observed in SFV4-infected HOS and A549 cells (Fig. 2n). LC3-I to LC3-II conversion was observed in both HOS and A549 cells with time post-SFV4 infection, and SQSTM1/p62 levels decreased in HOS cells (Fig. 2o, Supplementary Fig. 3g–j) at early phase of infection and decreased in A549 overtime during infection, indicating that the cargo in the autophagosome were degraded. However, the p62 level were restored in the late phase of infection of HOS cells. Vacuolization of the cytoplasm was not observed in SFV4-infected cells, rather stress granule-like structures were present (Supplementary Fig. 5d). SFV4 replication centers, detected by staining for dsRNA co-localized with LC3-II, suggesting that LC3-II is present in SFV4 replication complexes during viral RNA replication (Supplementary Fig. 6a–c). Autophagy was initiated upon SFV4 infection whilst vacuolization of cytoplasm was not observed. In conclusion, our results indicate that SFV4 induces rapid cell lysis accompanied by induction of apoptosis.

### Vaccinia virus-mediated cell lysis is accompanied by induction of necroptosis and autophagy

**Fig. 3:**
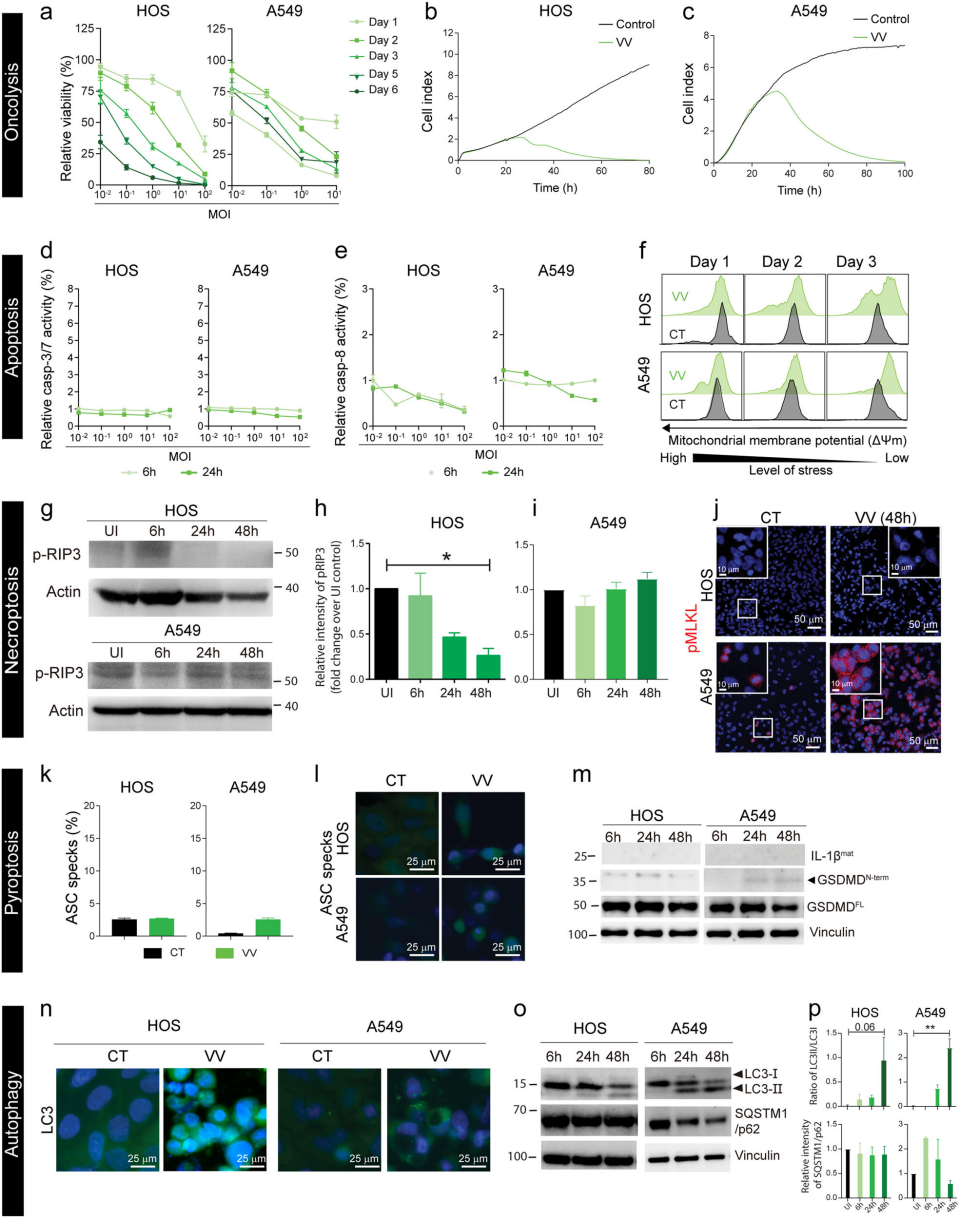
VV-induced cell death in HOS and A549 cells. *Oncolysis:* (**a**) Cell viability of VV-infected cells (MOI 10^-2^–10^2^) at days 1, 2, 3, 5, and 6 was measured using AlamarBlue™ viability assay. Cell viability is represented as percentage relative to non-infected control cells. Data are presented as mean ± SEM (*n* = 3). (**b**) HOS cells and (**c**) A549 cells were infected with VV at MOI 10. Cell index values as a measure of cell viability were measured by the xCELLigence system. *Apoptosis:* Analysis of (**d**) Caspase-3/7 and (**e**) Caspase-8 in VV-infected (MOI 10^-2^-10^2^) HOS and A549 cells at 6 h and 24 h was performed using Caspase-3/7ApoTox-Glo™ Triplex and Caspase-Glo® 8 assays. Caspase activity are presented as percentage relative to non-infected control cells. Data are expressed as mean ± SEM (*n* = 3). (**f**) Mitochondrial membrane potential was measured by flow cytometry after infection with VV (MOI = 10) at days 1, 2 and 3. *Necroptosis:* (**g**) Phosphorylated RIP3 (p-RIP3) was detected in VV-infected (MOI 10) HOS and A549 cells by Western blot 6, 24 and 48 h after infection. Densitometric analysis of fold change in p-RIP3 post VV-infection in (**h**) HOS and (**i**) A549 compared to un-infected control (*n* = 3). (**j**) HOS and A549 cells cultured on glass slides were infected with VV (MOI = 10) for 48 h and phosphorylated MLKL (p-MLKL) was detected by antibody staining (red). Cell nuclei were stained with Hoechst 33342 (blue). The multicolor fluorescent analyses were carried out in three individual experiments. Representative images from one experiment are shown. *Pyroptosis:* HOS and A549 cells expressing GFP-ASC were infected with the VV (MOI 10) for 48 h. ASC specks were detected by flow cytometry (**k**) and representative images (**l**) were taken from cells cultured on glass chamber slides. (**m**) Lysates were harvested from VV-infected HOS and A549 cells (MOI = 10) at 6, 24 and 48 h. Full-length GSDMD^FL^, truncated and active GSDMD^N-term^ (lower band, indicated by arrow) and mature IL-1β^mat^ were detected by Western blot. Vinculin was used as internal loading control. *Autophagy:* HOS and A549 cells expressing eGFP-LC3B cells were infected with VV (MOI = 10) and monitored by fluorescence microscopy. (**n**) Images were acquired at 48 h. (**o**) The cell lysates of VV-infected HOS and A549 cells (MOI = 50) were harvested at 6, 24, and 48 h for analysis of the non-lipidated form of LC3 (LC3-I), lipidated form (LC3-II) and the cargo-loading adaptor protein SQSTM1/p62. The housekeeping membrane-cytoskeletal protein vinculin was used as gel loading control. (**p**) Densitometric analysis of ratio of LC3-II/LC3-I and SQSTM1/p62 degradation VV-infection in HOS and A549 compared to un-infected control. Statistical analysis for normally distributed data were performed by means of one-way ANOVA with Dunnet’s post hoc test and for not normally distributed data were performed by means of Kruskal–Wallis test with Dunn’s post hoc test (*p < 0.05, **p < 0.01 and *n* = 3).

*Oncolysis:* Vaccinia virus (VV) Western Reserve infection led to rapid cell death in both cell lines in a dose and time-dependent manner (Fig. 3a), and the results were confirmed by xCELLigence measurements (Fig. 3b, c). *Apoptosis:* Caspase-3/7 or caspase-8 activation was not observed upon VV infection (Fig. 3d, e) and if anything, caspase-8 activity rather seemed to decrease (Fig. 3e). However, VV infection of HOS cells lead to decrease in mitocondrial membrane potential Δψm overtime (Fig. 3f). The above results indicate that VV infection is accompanied by an increase in mitochondrial stress but not in functional apoptosis. *Necroptosis:* Uninfected HOS and A549 cells had very low p-RIP3 and p-MLKL. VV infection of HOS cells reduced phosphorylation of RIP3 overtime and p-MLKL was not detected, indicating that VV did not trigger necroptosis in HOS cells (Fig. 3g, h, j). No change in phosphorylation of RIP3 was observed post-VV-infection in A549 cells (Fig. 3g–i, Supplementary Fig. 3a, b) but, a marked increase of p-MLKL was observed in A549 upon VV-infection (Fig. 3j). These results taken together with decrease in caspase-8 activity (Fig. 3e) suggests activation of necroptosis upon VV-infection in A549 cells. Necroptosis is a cell death modality previously reported to be triggered by VV^18^. *Pyroptosis:* Increase of ASC specks were not detected after VV-infection, as compared to uninfected A549-GFP-ASC or HOS-GFP-ASC cells (Fig. 3k, l, Supplementary Fig. 4b, e). Mature IL-1β^mat^ (17-kDa) was absent in VV-infected HOS and A549 (Fig. 3m, Supplementary Fig. 3c, d). However, low levels of cleaved GSDMD^N-term^ was detected after VV-infection in A549 at 24 and 48 h p.i. but not in HOS cells (Fig. 3m, Supplementary Fig. 3e, f). These results suggest that inflammasomes are not formed upon VV infection although the pyroptosis execution molecule GSDMD^N-term^ is present in VV-infected A549 cells. *Autophagy:* VV-infected HOS did not have LC3 punctate structures, but A549 had a few LC3 punctate structures in the cytoplasm (Fig. 3n). Pronounced LC3-I to LC3-II conversion was observed overtime post-VV-infection in A549 cells (Fig. 3o, Supplementary Fig. 3g). Degradation of SQSTM1/p62 was only observed in VV-infected A549 cells at 48 h post-infection (Fig. 3o, Supplementary Fig. 3j). This indicates that autophagy was initiated at least in A459 cells upon VV infection whilst vacuolization of cytoplasm was not observed during VV infection (Supplementary Fig. 5e). In conclusion, VV-mediated cell lysis is primarily accompanied by induction of necroptosis and autophagy in A549 cells.

### Ad- and SFV- but not VV-infection of tumor cells, induce potent ICD, with subsequent activation of DCs and antigen-specific T-cells

**Fig. 4:**
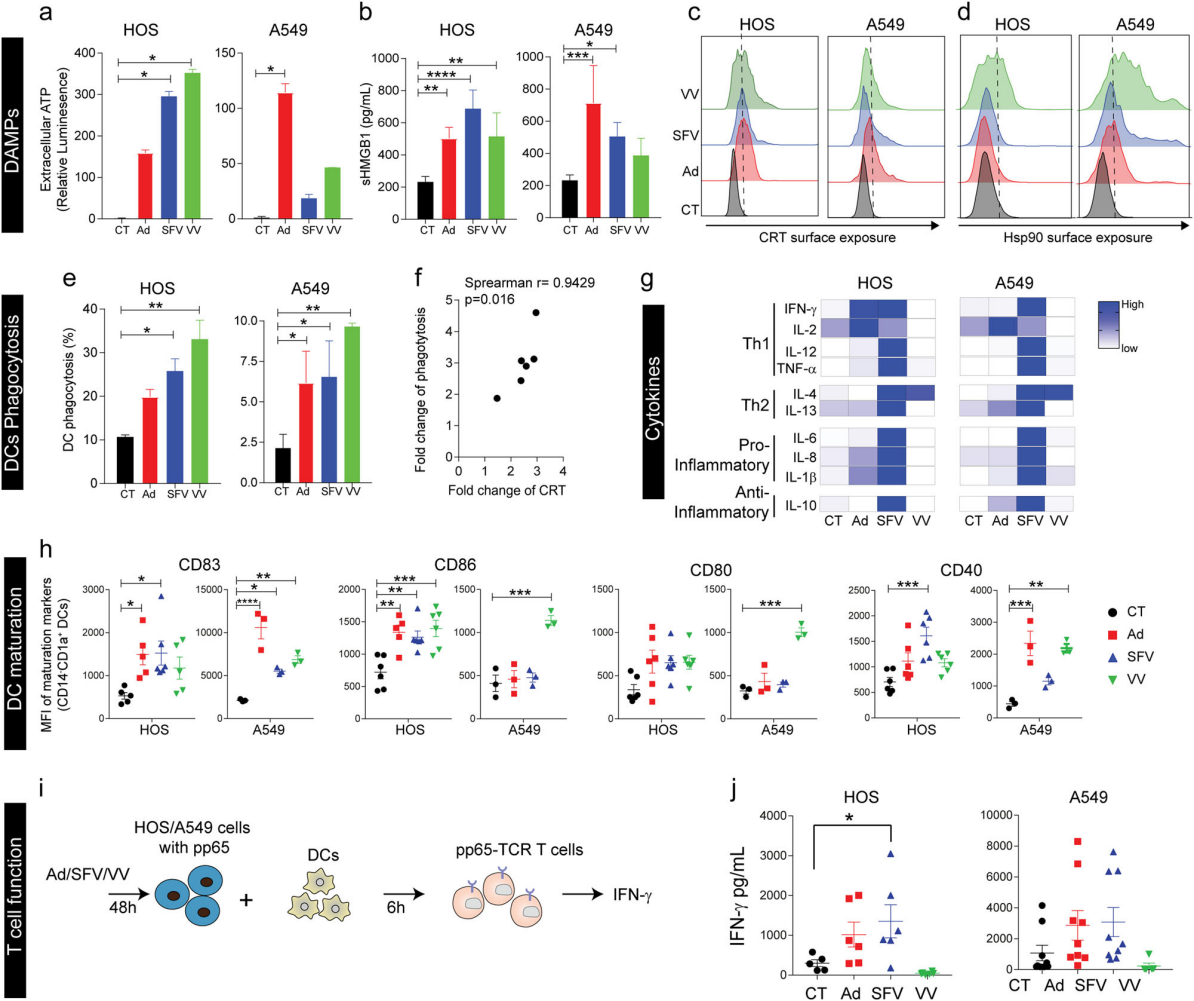
Virus-induced immunogenic cell death can activate DCs capable of stimulating antigen-specific T-cell responses. Experiments were performed using Ad, SFV4, and VV-infected HOS and A549 cells (MOI = 10) at 48 h. *DAMPs:* (**a**) Extracellular ATP (relative luminescence) was measured and represented as percentage relative to non-infected control (CT) cells. **b** Released HMGB1 was measured by ELISA. Calreticulin (CRT) exposure (**c**) and HSP90 expression (**d**) were measured by flow cytometry. *DC phagocytosis:* pp65-copGFP-expressing HOS cells (HOS-copGFP-pp65) and A549 cells (A549-copGFP-pp65) were infected with Ad, SFV4 or VV for 48 h. The infected cells were collected, washed and co-cultured with immature DCs at a ratio of 1:1 for 2 h before flow cytometry analysis. The population of CD1a^+^GFP^+^ double-positive cells were determined as the proportion of phagocytosis by DCs (**e**) and the correlation between fold change of phagocytosis and fold change of CRT (**f**). *Cytokine release:* (**g**) Supernatants from the co-cultures were collected after 24 h and Th1, Th2, pro- and anti-inflammatory cytokines were measured by Mesoscale. *DC maturation:* (**h**) After 48 h of co-culture, DC maturation was examined in terms of upregulation of the co-stimulatory molecules CD83, CD86, CD80 and CD40. *T-cell priming:* (**i**) Illustration of the experimental set-up. T-cells genetically engineered with a TCR specific for an HLA-A2-restricted epitope of pp65 (pp65-TCR T-cells) were stimulated for 6 h with autologous DCs that had been co-cultured for 48 h with Ad-, SFV4- and VV-infected HOS-copGFP-pp65 and A549-copGFP-pp65 cells, respectively. Uninfected cells were used as control (CT). **j** IFN-γ was measured by means of ELISA after 16 h. All data are expressed as mean ± SEM and performed with at least three different donors. Statistical analysis for normally distributed data were performed by means of one-way ANOVA with Bonferroni post hoc test and for not normally distributed data were performed by means of Kruskal–Wallis test with Dunn’s post hoc test (*p < 0.05, **p < 0.01, ***p < 0.001 and **** < 0.0001).

*DAMPs:* Release of bona fide ICD markers upon virus infection were measured. All three viruses triggered release of extracellular ATP upon infection, although the amounts varied depending on the virus and cell line (Fig. 4a). SFV4 and VV induced more ATP release from HOS, while Ad induced more ATP release from A549 compared to respective controls (Fig. 4a). This difference could be attributed to the greater abilities of Ad to kill A549, and SFV4 and VV to kill HOS (Figs. 1a–c, 2a–c, 3a–c). An increase of extracellular HMGB1 was detected in the supernatant collected from HOS and A549 cells infected with each of the viruses, although not significant for VV in A549 (Fig. 4b). All viruses also caused increase in surface exposure of calreticulin (CRT) except for SFV4 in A549 (Fig. 4c). Surface exposure of CRT was confirmed with imaged based flow cytometry (Supplementary Fig. 7). An increase in surface exposure of HSP90 was detected on HOS after VV infection, but not after Ad or SFV4 infection (Fig. 4d), while all three viruses induced surface exposure of HSP90 in A549 (Fig. 4d).

Next, we analyzed the ability of virus-mediated cell death to stimulate DCs to activate antigen-specific T-cells. *Phagocytosis:* DC ingestion of virus-infected pp65-copGFP-expressing tumor cells was measured. Phagocytosis of virus-infected cells, as detected through copGFP uptake, was significantly enhanced for all viruses except for Ad-infected HOS where a trend of increased phagocytosis was observed (Fig. 4e, Supplementary Fig. 8). Importantly, phagocytosis correlated with the presence of CRT on the surface of virus-infected cancer cells (Fig. 4f). *Cytokine release:* Cytokine release by DCs co-cultured with virus-infected tumor cells was measured. Only SFV4-infected tumor cells consistently induced DCs to secrete Th1 cytokines (IFN-γ, IL-12, TNF-α), Th2 cytokines (IL-4, IL-13), proinflammatory cytokines (IL-6, IL-8, IL-1β,) and anti-inflammatory cytokine (IL-10) (Fig. 4g, Supplementary Fig. 9). Ad-infected tumor cells partly stimulated DCs to secrete Th1 cytokines, whereas, VV-infected tumor cells seemed to suppress cytokine secretion by DCs compared to uninfected tumor cells (Fig. 4g, Supplementary Fig. 9). *Phenotypic maturation and activation:* The CD83 maturation marker was upregulated on DCs when co-cultured with virus-infected tumor cells (Fig. 4h). The activation status of DCs, assessed as upregulation of CD86, CD80 and CD40 when co-cultured with virus-infected tumor cells, was both virus and cell line-dependent, with some degree of activation in all cases (Fig. 4h). In addition, we verified that direct infection of DCs did not promote DC maturation (Supplementary Fig. 10a–d), indicating that activation of DCs is due to virus-induced ICD of infected cells. *Antigen processing and cross-presentation:* The capacity of DCs to cross-present phagocytosed antigen to CD8^+^ T-cells was evaluated using CMV-pp65 as a model antigen. Human T-cells engineered to express a TCR specific for the HLA-A0201-restricted pp65_495-503_ peptide^19^, pp65-TCR T-cells, were co-cultured with autologous DCs, previously exposed to virus-infected copGFP-pp65-expressing tumor cells (Fig. 4i). There was a significant increase in IFN-γ secretion by pp65-TCR T-cells when they encountered DCs co-cultured with SFV4-infected HOS-copGFP-pp65 cells (Fig. 4j), indicating that DCs could take up tumor-associated antigens (in this case the pp65 model antigen) from SFV4-infected and dying tumor cells and cross-present antigenic peptides to activate antigen-specific CD8^+^ T-cells. There was a trend towards increased IFN-γ secretion by pp65-TCR T-cells when encountered DCs co-cultured with Ad-infected HOS but not with VV-infected HOS (Fig. 4j), which may be due to inhibition of immune response by VV-encoded genes. There was a clear trend that DC uptake of both Ad- and SFV4-infected A549 leads to activation of antigen-specific T-cell. The reason why significance was not reached we believe is because A549 is HLA-A2 positive and the cells can directly present the pp65 epitope to T-cells and activate them, as indicated by high levels of IFN-γ when non-infected A549 were used as stimuli (Fig. 4j). In conclusion, SFV4 is the most immunogenic of the three viruses, closely followed by Ad, while VV has the ability to counteract immunogenicity in the examined tumor cell lines.

## Discussion

In spite of the successful development of Ad and VV as oncolytic agents in the clinic and SFV4 preclinically, a comprehensive description and comparison of the cell death induced by these viruses and their contribution to elicit an anti-tumor immune response is largely unknown. Virus tropism results in cell type-specific activities, which also results in differences in the induced immune response. Adenoviruses serotype 5 is known to naturally cause infection in the upper respiratory tract system^20^, which may explain its ability to kill the human lung adenocarcinoma cell line A549 better than the human osteosarcoma cell line HOS (Fig. 1a–c). SFV4 and VV are capable of infecting and killing both cell types (Fig. 2a–c and Fig. 3a–c). It should be noted that wild-type viruses were used in this study and that their OV counterparts, which carry genetic modifications can have different infection and replication kinetics. Therefore, when developing an OV for an indication it is important to consider the characteristics of the virus as it can affect its replication and the resulting mode of cell death initated during viral oncolysis can differ considerably. The mode of cell death intiated can dictate the induced anti-tumor immune response, as summarized in Table 1.

**Table 1:** Summary of Adenovirus, Semliki Forest Virus and Vaccinia Virus-induced immunogenic cell death (ICD).

Virus	Cell Death Modality				ICD and DAMPs	DC activation	T cell activation (IFN- γ)
	Apoptosis	Necroptosis	Pyroptosis	Autophagy			
**Adenovirus** • Small dsDNA • Non-enveloped • Replicates well in A549	• No Caspase-3/7 or Caspase-8 stimulation • No change in mitochondrial potential	• Induced p-RIP3 and p-MLKL	• Inflammasome assembly and mature IL-1β • No GSDMD cleavage	• Autophagosome formation • LC3-I lipidation • Degradation of SQSTM/p62 • Vacuolization of the cytoplasm observed	• ATP release in A549 • HMGB1 release • CRT surface exposure • HSP90 surface exposure in A549	Cytokines •**Th1:** IFN-γ, IL-1β, IL-2 Activation markers •CD83, CD86, CD40	Trend towards activation of antigen-specific T-cells
**Semliki Forest virus** • Small ssRNA (+) • Enveloped • Replicates well in HOS	• Activation of Caspase-3/7 and Caspase-8 • Decrease in mitochondrial potential	• Decrease in p-RIP3 levels • No change in p-MLKL	• No inflammasome assembly or mature IL-1β • GSDMD cleavage in HOS	• Autophagosome formation • LC3-I lipidation • Degradation of SQSTM/p62 in A549 • No vacuolization of the cytoplasm	• ATP release in HOS • HMGB1 release • CRT surface exposure • HSP90 surface exposure in A549	Cytokines • **Th1:** IFN-γ, IL-1β, IL-12, IL-2, TNF-α • **ProInflammatory:** IL-6, IL-8 • **Th2-** IL-4, IL-13 • **Anti-inflammatory:** IL-10 Activation markers • CD83, CD86, CD40	Significant activation of antigen-specific T-cells
**Vaccinia virus** • large dsDNA • Enveloped • Replicates well in both A549 and HOS	• No Caspase-3/7 or Caspase-8 stimulation • Partial decrease in mitochondrial potential	• Marginal increase in p-RIP3 in A549 • Increase in p-MLKL levels in A549	• No inflammasome assembly or mature IL-1β • Low levels of GSDMD cleavage in A549	• No pronounced autophagosome formation • LC3-I lipidation • Degradation of SQSTM/p62 in A549 • No vacuolization of the cytoplasm	• ATP release in HOS • HMGB1 release • CRT surface exposure • HSP90 surface exposure	Cytokines • Inhibit most Th1 cytokine secretion • **Th2:** IL-4 Activation markers • CD83, CD86, CD40	Inhibits T-cell activation

In general, the tested viruses triggered multiple programmed cell death pathways and viral oncolysis was observed despite inhibiting apotosis, necroptosis, pyroptosis or autophagy (Supplementary Fig. 11). Ad-infection triggers oncolysis along with induction of multi-modal cell death mechanisms involving necroptosis and pyroptosis signaling cascades in the presence of a sustained autophagic activity. Ad is well known for its ability to induce autophagy in tumor cells^21,22^. Autophagy plays a critical role in eliminating viral infection, by degrading viruses and viral components in the autophagolysosome^23^. It is also known that autophagy induction, upon viral infection, initiates the IFN-I responses by delivering virus-derived PAMPs to pattern recognition receptors^24^, which can be detrimental for virus propagation. On the other hand, induction of type-I IFNs by Stimulator of Interferon Genes (STING) signaling, especially in DCs, can be very beneficial for priming of anti-tumor T-cell responses^25^. In addition, cancer cells undergoing either pyroptosis or autophagy are known to facilitate recruitment of and phagocytosis by APCs, presumably through ATP release, and phosphatidylserine exposure^26,27^, which are good prognostic factors for successful immunotherapy.

SFV4-mediated cell lysis is accompanied by activation of apoptosis, in agreement with previous findings^28^. Death by physiological apoptosis is considered non-immunogenic^29^. However, recent findings strongly indicate that certain cytotoxic drugs (e.g. doxorubicin, oxaliplatin)^30,31^ and radiation^32^ can induce immunogenic apoptosis that activates APCs via TLR-2/TLR-9-MyD88 signaling^30^. SFV4-induced apoptosis is highly immunogenic as SFV4-infected tumor cells induced activation and maturation of DC to produce Th1 and proinflammatory cytokines and chemokines. SFV4-infected tumor cell material was phagocytosed by DCs and cross-presented for efficient activation of antigen-specific CD8^+^ T-cells.

VV-mediated cell lysis was accompanied by induction of necroptosis in A549 cells and autophagic events were observed at least in A549. VV-infection in fibroblasts and T-cells in known to induced RIP3-mediated necroptosis, sensitized by TNF-α^33^. Also, in another study VV-infection in ovarian cancer cells induced RIP3-mediated necroptosis, but this was not dependent on autocrine secretion of TNF-α^18^. Therefore, the exact mechanism by which VV triggers necroptosis is still a question that needs to be further investigated.

ICD involves changes in the composition of the cell surface of dying cells and the release of soluble DAMPs in a defined time sequence. ATP secretion and CRT surface exposure is followed by HMGB1 and HSP secretion at later stages^34^. In addition, virus infection can lead to accumulation of PAMPs, including viral proteins and nucleic acids, which are potent stimulators of the immune system^2,35^. All three viruses triggered release of ICD-characteristic DAMPs, i.e., ATP, HMGB1, CRT, and HSP90 (Fig. 4a–d). The ability of the viruses to induce DAMPs depended on their ability to infect and replicate in a particular tumor cell type. For example, Ad killed A549 cells better than it killed HOS cells, correlating with higher ATP, HMGB1 and HSP90 release, and CRT exposure (Fig. 4a–d). Extracellular ATP activates the NLRP3-ASC-inflammasome axis leading to secretion of mature IL-1β^36,37^. Ad-infection is associated with sustained autophagy (Fig. 1n, o), a pathway known to induce ATP release and inflammasome activation^37,38^, which explains the observed inflammasome activation (Fig. 1k, l) and mature IL-1β secretion (Fig. 1m) after Ad-infection. CRT exposure dictates the immunogenicity of a dying cancer cell, as it serves as an “eat me” signal for phagocytic cells such as DCs, thereby promoting phagocytosis of tumor-associated antigens from virus-killed tumor cells and subsequent cross-presentation to and activation of antigen-specific cytotoxic T-cells^39^. During virus-induced cell death, we observed CRT exposure and subsequent phagocytosis of the dying cells by immature DCs (imDCs) (Fig. 4c, e, f). At later stages of cell death, HSPs (70/90) and nuclear protein HMGB1 are released. HMGB1 binds to receptors (TLR2, TLR4, TLR9, TIM3 and RAGE) on APCs to initiate a potent proinflammatory cytokine response^40–42^. ImDCs were activated to release significantly higher amounts of cytokines only when co-cultured with SFV4-infected tumor cells (Fig. 4g). On the contrary, when imDCs were co-cultured with VV-infected tumor cells, they did not secrete any proinflammatory cytokines (Fig. 4g, Supplementary Fig. 9). It should be noticed that the VV-encoded proteins A52, B15, and K7 are inhibitors of NF-kB and known to suppress chemokine/cytokine expression from immune cells^43^. Ad- and SFV4-mediated DC activation is immune stimulating as assessed by release of IFN-γ from model antigen-specific CD8^+^ T-cells. Although VV-infected tumor cells exhibited signs of ICD and such cells were efficiently phagocytosed by DCs. However, these DCs failed to promote T-cell responses (Fig. 4j). We did not observe DC or T-cell death upon VV-infection (data not shown), ruling it out as a possible reason for the observed absence of T-cell priming (Fig. 4j). VV is known to evade immune response by expressing decoy receptors to neutralize immune stimulatory cytokines including type-I IFNs and IL-1β^44,45^. VV-infected DCs are also malfunctioning and have difficulty to elicit T-cell responses^46,47^. Importantly, DCs phagocytosing VV-infected tumor cells, failed to induce T-cell responses (Fig. 4j). On the other hand, attenuated VV activates STING and Batf3 pathways in DCs and subsequently induce potent anti-tumor immunity^48^. Therefore, VV inactivation may be a possible strategy to subvert VV-induced immune suppression.

The immunogenicity of virus-induced cell death mechanisms can be expected to have impact on the immunotherapeutic effect. Virus-mediated cancer cell lysis leads to local release of host-derived DAMPs (that can depend on the type of cell death pathways activated upon virus infection), virus-derived PAMPs, as well as viral and tumor antigens, together leading to ICD. Therefore, utilizing viruses as therapeutic agents may enhance recruitment of immune cells to the tumor microenvironment and cause efficient cross-priming of TAAs including neoantigens to T-cells, as indirectly suggested by clinical observations^7^. However, as an evolutionary survival mechanism^9^ viruses (e.g. VV in our study) may also interfere with the host cells to avoid the antiviral immune response, thus suppress also the anti-tumor immune response. Therefore, OVs that (either naturally or due to genetic modifications) do not have the ability to interfere with ICD pathways would be beneficial in order to increase the chances of mounting an effective anti-tumor immune response. We found that SFV4 was especially appropriate in this sense. Overall, OVs are attractive drugs especially against immunologically cold tumors, where virus-induced ICD can convert the otherwise nonresponsive microenvironment responsive to immunotherapy^49–51^.

## Materials and methods

### Cell lines and culture conditions

The human bone osteosarcoma cell line HOS (ATCC® CRL-1543™) and lentivirus-engineered HOS-derived lines (HOS-GFP-ASC, HOS-eGFP-LC3B, HOS-copGFP-pp65) were cultured in Dulbecco’s modified Eagle’s medium (DMEM) with 10% heat-inactivated fetal bovine serum (FBS), 100IU/ml penicillin and 100 μg/ml streptomycin (1% PEST), and 1 mM sodium pyruvate. The human lung carcinoma cell line A549 (ATCC® CCL-185™) and lentivirus-engineered A549-derived lines (A549-GFP-ASC, A549-eGFP-LC3B, A549-copGFP-pp65) were cultured in Roswell Park Memorial Institute (RPMI)-1640 medium supplemented with 10% FBS, 1% PEST, and 1 mM sodium pyruvate. All components and culture media were from Thermo Fisher Scientific (Waltham, MA). All cells were cultured in a humidified incubator with a 5% CO_2_ atmosphere at 37 °C.

### Viruses

Wild-type human Adenovirus serotype 5 (Ad) was obtained from ATCC and was amplified and purified as described previously^11^. Semliki Forest virus (SFV) strain 4 was obtained from Andres Merits, University of Tartu, Tartu, Estonia and was amplified and purified as previously described^15^. Vaccinia virus (VV) Western Reserve stain was obtained from Bernard Moss, National Institute for Allergy and Infectious Diseases, and was amplified and purified as described previously^52^. Viruses were stored at −80°C. Ad titers were based on fluorescent-forming units (FFU) while SFV4 and VV titers were based on plaque-forming units (PFU). All the multiplicity of infection (MOI) used in this study is based on PFU/cell for SFV4 and VV, and FFU/cell for Ad.

### OV cytotoxicity assays

#### AlamarBlue cell viability assay

HOS cells and A549 cells (1 × 10^4^ cells/well, 96-well plates) were infected with Ad, SFV4 or VV at MOIs ranging from 100 to 0.01. (*n* = 3, biological replicates for each MOI). Cell viability was measured using AlamarBlue™ viability reagent (Thermo Fisher Scientific) 1, 2, 3, 5, and 6 days post-infection (p.i.). Signals were monitored by fluorometry with excitation at 530 nm and emission at 590 nm in a Synergy HTX Multi-Mode multiplate reader (BioTeck Instruments Inc., Winooski, VT).

#### xCELLigence cell viability assay

HOS cells and A549 cells (10,000/well) were infected with Ad, SFV4 or VV at MOI 10 in 96-well electronic microtiter plate (ACEA Biosciences, San Diego, CA). Infected cells were incubated at 37 °C and real-time cell viability was monitored as cell index using the xCELLigence RTCA SP instrument (ACEA Biosciences).

### Apoptosis assays

#### Caspase activity measurements

Cells (1 × 10^4^ cells/well, 96-well plates) were infected with viruses at MOI 10 for 6 h or 24 h (*n* = 3). Caspase activity was measured using ApoTox-Glo™ Triplex (caspase-3/7) and Caspase-Glo-8 assay kits (Promega, Madison, WI). Luminescence signal was measured using a Synergy HTX Multi-Mode reader and the data was normalized by background subtraction and in relation to uninfected cells (*n* = 3, biological replicates). Mitochondrial membrane potential (ΔΨm) assay: Cells were infected with viruses at an MOI 10 for 48 h. The MitoTracker™ Deep Red FM kit (Thermo Fisher Scientific) was used to measure mitochondrial membrane potential (*n* = 3, biological replicates).

### Necroptosis assay

#### Immunoblotting of phosphorylated receptor-interacting protein kinase 3 (p-RIP3) and of total and phosphorylated mixed-lineage kinase domain-like pseudokinase (MLKL)

Virus-infected HOS and A549 cells (MOI 10) were harvested (*n* = 3, biological replicates), washed in saline and lysed in RIPA buffer supplemented with Halt™ protease and phosphatase inhibitors (Thermo Fisher Scientific). Proteins in the lysates were denatured by heating at 95 °C for 10 min. Equal amounts of proteins were separated using 4–12% Bis-Tris NuPAGE™ gel (Thermo Fisher Scientific), transferred to a nitrocellulose membrane and probed with antibodies. Specific protein bands were visualized using ECL™ Western Blotting Detection reagents (GE Healthcare Life Science, Uppsala, Sweden) and images were obtained by using ChemiDoc MP (Biorad, Hercules, CA). Rabbit monoclonal anti-RIP3 (phosphor S227) antibody (clone: ab209384, Abcam, Cambridge, MA) was used to detect phosphorylated RIP3. The intensity of specific bands were quantified with Image J (National Institutes of Health, USA), normalized against beta-actin that was detected by monoclonal antibody (clone AC-15, Sigma Aldrich, Germany) and fold change to the uninfected controls were calculated. *Staining of phosphorylated MLKL:* HOS and A549 cells were cultured on glass chamber slides and infected with viruses at MOI 10 for 48 h. Cells were then fixed with 4% formaldehyde, permeabilized with 0.05% Triton-X100 and blocked with 4%BSA TBS-T for 1 h, washed with TBS-T and stained with a rabbit monoclonal anti-phosphoS358-MLKL antibody (clone: ab187091, Abcam) for 2 h at room temperature and then probed with a donkey anti-mouse Alexa-fluor647 secondary antibody (Thermo Fisher Scientific). Nucleus was stained with Hoechst 33342 (Thermo Fisher Scientific) and images were obtained using a Leica SP-8 confocal microscope (Leica microsystems).

### Pyroptosis assays

#### Measurement of apoptosis-associated speck-like protein containing a CARD (ASC) specks

HOS-GFP-ASC and A549-GFP-ASC were infected with the virus at MOI 10 for 48 h. ASC specks were detected by flow cytometry^53^ and images were taken from cells cultured on glass chamber slides. *Immunoblotting of full-length gasdermin-D (GSDMD*^*FL*^*), the active N-terminal fragment of GSDMD (GSDMD*^*N-term*^*) and mature IL-1β (IL-1β*^*mat*^*):* Virus-infected cells (MOI 10 for 6 to 48 h) were prepared for Western blot as described above (IL-1β^mat^, *n* = 3, GSDMD^N-term^, *n* = 2, biological replicates). Antibodies used: rabbit polyclonal anti-GSDMD (clone: NBP2-33422, Novus Biologicals), rabbit polyclonal anti-IL-1β (clone: ab53175, Abcam), and rabbit monoclonal anti-vinculin (clone: 700065, Thermo Fisher Scientific).

### Autophagy assays

#### Microtubule-associated proteins 1A/1B light chain 3 (LC3) location assay

HOS-eGFP-LC3 and A549-eGFP-LC3 cells were infected with viruses at MOI 10 for 48 h (*n* = 3, biological replicates). LC3 accumulation in autophagosomes were visualized in an eclipse Ti-S inverted microscope system (Nikon, Tokyo, Japan) equipped with a Zyla sCMOS camera (Andor Technology, Belfast, Northern Ireland).

#### Immunoblotting of LC3 and sequestosome-1 (SQSTM1)/p62

cells were infected with viruses at MOI 10 and cell lysates were harvested at various time points (HOS cells, *n* = 3, A549 cells, *n* = 2, biological replicates). Western blot was performed as described above. The primary antibodies used were: rabbit polyclonal anti-LC3B (clone: PA1-16930, Thermo Fisher Scientific) and rabbit polyclonal anti-SQSTM1/p62 (clone: 5114, Cell Signaling Technology, Danvers, MA). The goat anti-rabbit IgG(H + L)-HRP conjugated antibody (clone: G-21234, Thermo Fisher Scientific) was used as the secondary probe. The intensity of the LC3-I and LC3-II bands was quantified with Image J

### Analysis of DAMPs

HOS and A549 cells were infected with viruses at MOI 10 for 48 h. *Extracellular ATP:* Supernatants were collected after 48 h and extracellular ATP was measured with an ATP Detection Kit (Thermo Fisher Scientific) (*n* = 2, biological replicates). *HMGB1 release:* HMGB1 was measured with ELISA kit (Nordic BioSite, Sweden) from supernatant of virus-infected cells (*n* = 4, biological replicates). *Calreticulin (CRT) exposure:* Infected cells were stained with the anti-calreticulin antibody (Catalog # PA3-900, Thermo Fisher Scientific) and secondary monkey anti-rabbit IgG-Alexa-Fluro633 (Thermo Fisher Scientific) (*n* = 4, biological replicates, one representative histogram is shown in Fig. 4c). *HSP90 exposure:* Infected cells were stained with primary anti-HSP90 beta antibody (Catalog # PA5-26001, Thermo Fisher Scientific) and secondary monkey anti-rabbit IgG-Alexa-Fluro647 (Thermo Fisher Scientific) (*n* = 3, biological replicates, one representative histogram is shown in Fig. 4d). The samples were analyzed by BD Canto II flow cytometer (BD Biosciences, Stockholm, Sweden).

### Isolation of human PBMCs and generation of DCs

Buffy coats were obtained from the blood center at the Uppsala University Hospital. Peripheral blood mononuclear cells (PBMCs) were isolated from fresh buffy coats by Ficoll-Paque separation (GE Healthcare Life Science). PBMCs were cultured in culture medium (RPMI-1640 consisting of 10% FBS, 1% PEST, 2mM l-glutamine, 1 mM HEPES and 20 mM β-mercaptoethanol). Monocytes were obtained by CD14^+^ magnetic beads isolation (Miltenyi Biotec, Lund, Sweden) and differentiated to immature DCs (imDCs) in culture medium containing 20 ng/ml human IL-4 (Gentaur, Brussels, Belgium) and 100 ng/ml GM-CSF (Gentaur) for 5 days. The medium was replaced with fresh culture medium supplemented with GM-CSF and IL-4 every 2 days.

### Functional immunology assays

#### Phagocytosis assay

HOS-copGFP-pp65 and A549-copGFP-pp65 were infected with viruses at MOI 10 for 48 h, washed and co-cultured with monocyte-derived DCs at a ratio of 1:1 for 2 h (HOS *n* = 5, A549 *n* = 3, biological replicates). The co-cultured DCs were stained with PerCP/Cy5.5-anti-CD1a (BioLegend) and analyzed by flow cytometry. True phagocytosis of the copGFP-labeled dead cells by DCs was determined by using a gating strategy that allows analysis of only CD1a^+^GFP^+^ (double-positive) cells. *DC maturation*: HOS and A549 cells were infected with viruses at MOI 10 for 48 h. The infected cells were collected, washed in culture medium and co-cultured with 2 × 10^5^ imDCs in a ratio 1:1 for 48 h. Maturation of DCs was analyzed by flow cytometry. Antibodies used to detect maturation markers were BV510-anti-CD1a, APC-anti-CD80, PE-Cy7-anti-CD83, BV421-anti-CD86 and PE-anti-CD40. All antibodies were purchased from BD BioLegend (San Diego, CA). *Cytokine profiling***:** Cytokines were measured from co-cultures of virus-infected cells with imDCs by using the V-PLEX proinflammatory Panel 1 Human Kit (Meso Scale Diagnostics, Rockville, MD). *Antigen presentation assay in vitro***:** The experiment was performed as described previously^54^. Briefly, imDCs derived from monocytes of HLA-A*0201 positive blood donors were co-cultured with virus-infected HOS-copGFP-pp65 or A549-copGFP-pp65 to take up pp65. Autologous T-cells were transduced with a lentiviral vector expressing a T-cell receptor (TCR) for the HLA-A*0201-restricted cytomegalovirus (CMV) pp65_495-503_ peptide (NLVPMVATV), named pp65-TCR T-cells^19^, and cultured in culture medium with IL-2 (20 IU/mL) (Novartis, Basel, Switzerland). The pp65-TCR T-cells were added to autologous DCs co-cultured with virus-killed tumor cells in a ratio of 5:1 for 6 h. Supernatant from the cells were collected and used in a human interferon (IFN)-γ ELISA, according to the manufacturer’s protocol (Mabtech, Nacka Strand, Sweden) (HOS *n* = 5, A549 *n* = 9, biological replicates). Samples were excluded if the DCs were not viable after co-culture with virus-infected cells.

### Statistical analyses

The data are reported as means and SEM. Statistical analysis was performed by GraphPad Prism® software version 6.01 (La Jolla, CA). Two-sided statistical tests were performed with appropriate post hoc tests to adjust for multiple comparisons. Normal distribution of the data was assessed by Shapiro–Wilk normality test. Values with *p* < 0.05 were considered statistically significant. Detailed descriptions about statistical analysis are described in the figure legends.

## Supplementary information

Suppplementary Figure legends

supplementary figure 1

supplementary figure 2

supplementary figure 3

supplementary figure 4

supplementary figure 5

supplementary figure 6

supplementary figure 7

supplementary figure 8

supplementary figure 9

supplementary figure 10

supplementary figure 11
