# Supplementary material for: Characterization of virus-mediated immunogenic cancer cell death and the consequences for oncolytic virus-based immunotherapy of cancer
Source: Cell Death Dis. 2020 Jan 22;11(1):48. doi: 10.1038/s41419-020-2236-3 (PMC6976683; doi:10.1038/s41419-020-2236-3)
Supplement: Supplementary file 1 — Suppplementary Figure legends [file 41419_2020_2236_MOESM1_ESM.docx]

**Supplementary Figure Legends**

**Supplementary Figure 1. Illustration of the analyzed cell death pathways.** Apoptosis, necroptosis and pyroptosis are programmed forms of cell death. Autophagy is a survival pathway that if excessive or uncontrolled, promotes cell death. Abbreviations: Fas-associated protein with death domain (FADD); receptor-interacting protein kinase 1 (RIP1); receptor-interacting protein kinase 3 (RIP3); mixed lineage kinase domain-like (MLKL) protein; phosphorylated (p); danger-associated molecular patterns (DAMPs); pathogen-associated molecular patterns (PAMPs); NACHT, LRR and PYD domains-containing protein 3 (NLRP3); apoptosis-associated speck-like protein containing a caspase recruitment domain (ASC); gasdermin D (GSDMD); FL = full length; N-term = N-terminal; Interleukin 1 beta (IL-1β); Microtubule-associated protein 1A/1B light chain 3 (LC3); Sequestosome 1 (SQSTM1).

**Supplementary Figure 2. Ad5(GFP)-transduced HOS and A549 cells.** HOS **(a)** and A549 **(b)** cells were transduced with a non-replication competent adenoviral vector carrying the Green Fluorescent Protein transgene (Ad5(GFP)) at MOI 10, and analyzed by immunofluorescence microscope 48 hours later, demonstrating that both cell lines can be transduced by Ad5.

**Supplementary Figure 3. Western blots of whole gel images.** HOS and A549 cells were infected with Ad, SFV and VV respectively for 6h, 24h, 48h at MOI=10. **(a-b)** Phosphorylated RIP3 (p-RIP3) was detected in virus-infected HOS and A549 cells. **(c-d)** Mature IL-1β^mat^ were detected in virus-infected HOS and A549 cells. **(e-f)** Full-length GSDMD^FL^, truncated and active GSDMD^N-term^ (lower band, indicated by arrow) were detected in virus-infected HOS and A549 cells. **(g-h)** The LC3-I and LC3-II conversion were detected in virus-infected HOS and A549 cells. (**i-j**) SQSTM1/p62 were detected in virus-infected HOS and A549 cells. The box indicated cropped image used in main Figures 1-3.

**Supplementary Figure 4.** **The detection of ASC in infected HOS and A549 cells.** **(a)** The gating strategy of the detection of ASC specks flow cytometry. **(b-e)** HOS and A549 cells expressing GFP-ASC were left uninfected (CT) or were infected with the Ad, SFV and VV (MOI 10) for 48h. The representative large images were taken from cells cultured on glass chamber slides.

**Supplementary Figure 5. Electron microscopy pictures of HOS cells infected with viruses.** HOS cells were left uninfected (CT) (**a**) or were infected with Ad (**c**), SFV (**d**) and VV (**e**) for 48h at MOI 10. Positive control (Positive CT) (**b**) were obtained with cell treated by Ku0063794 (1uM) and BafA1 (10nM) for 24hrs. The red arrows indicated autophagsome formation in Ad-infected HOS cells (**c**).

**Supplementary Figure 6:** **Localization of autophagosomes with viral replication centers in SFV-infected HOS cells.** Images were acquired 48h after transduction. HOS cells expressing eGFP-LC3 (green) were infected with SFV (MOI=10) for 48h. Cells were co-stained for viral dsRNA (red) and nuclei with Hoechst 33342 (blue). Representative fluorescence microscopy images of **(a)** un-infected control cells, **(b)** SFV-infected cells and **(c)** zoom-in on SFV-infected cells.

**Supplementary Figure 7. Gating of DC phagocytosis.** The gating strategy for analysis of pp65-copGFP-expressing tumor cells phagocytosis by DCs (CD1a positive).

**Supplementary Figure 8. Cytokine release from imDCs co-cultured with virus-infected HOS and A549 cells.** Monocytes isolated from blood of five healthy donors were differentiated for 6 days into immature DCs (imDCs) with GM-CSF (100 ng/ml) and IL-4 (20 ng/ml). HOS and A549 cells were infected with Ad, SFV, and VV (MOI=10) for 48h. imDCs and virus-infected cells were co-cultured at a ratio of 1:1 for 48h. The co-culture supernatants were collected and **(a)** Th1 cytokines, **(b)** pro-inflammation cytokines and **(c)** Th2 cytokines were measured by Meso scale. Data are summarized and presented as heatmap in Figure 4g.

**Supplementary Figure 9. DC maturation is not induced by direct virus infection of the imDCs.** Monocytes isolated from blood of three healthy donors were differentiated for 6 days into immature DCs (imDCs) with GM-CSF (100 ng/ml) and IL-4 (20 ng/ml). They were then exposed to virus for 48h. DC maturation was examined in terms of upregulation of the co‑stimulatory molecule CD86 with a representative histogram figure **(a)** and average mean fluorescence intensity (MFI) **(b)**; and CD80 with a representative histogram figure **(c)** and average MFI **(d)**. As a positive control, DCs were matured by stimulation with R848 (2.5 µg/ml), IFN-γ (1000 IU/ml) and Poly I:C (20 µg/ml) for 48h and labelled mDCs in the diagrams.
