# Supplementary figures and images for: Characterization of virus-mediated immunogenic cancer cell death and the consequences for oncolytic virus-based immunotherapy of cancer
Source: Cell Death Dis. 2020 Jan 22;11(1):48. doi: 10.1038/s41419-020-2236-3 (PMC6976683; doi:10.1038/s41419-020-2236-3)

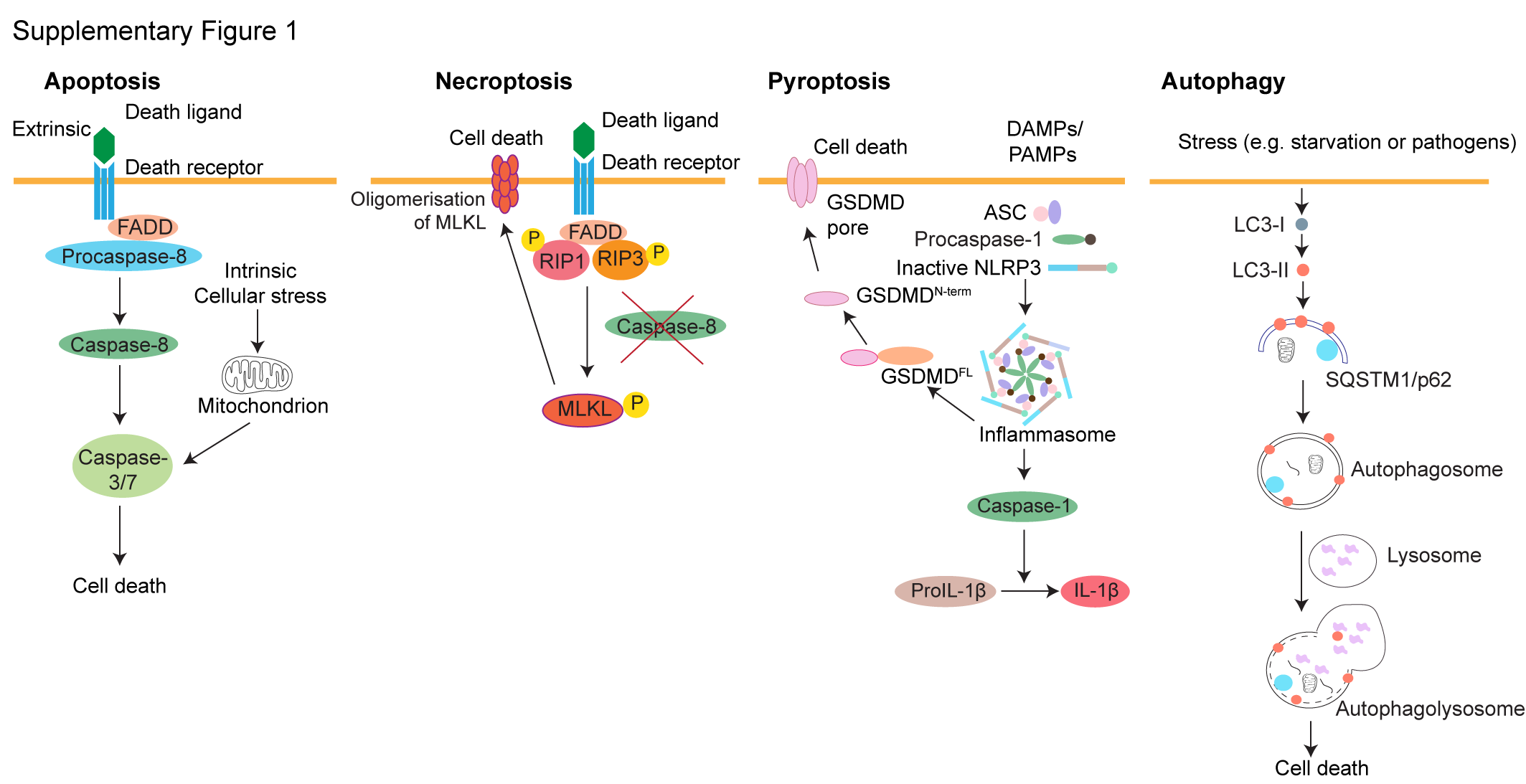

Supplement: Supplementary file 2 — supplementary figure 1 [file 41419_2020_2236_MOESM2_ESM.tif]

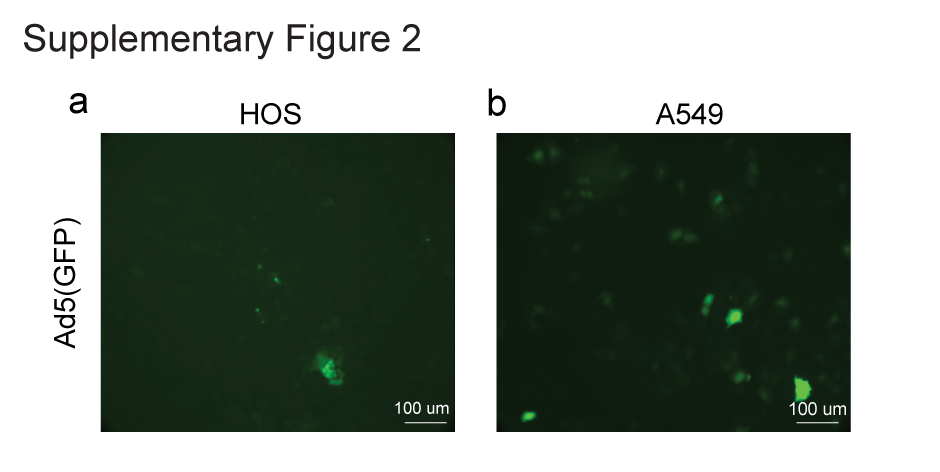

Supplement: Supplementary file 3 — supplementary figure 2 [file 41419_2020_2236_MOESM3_ESM.tif]

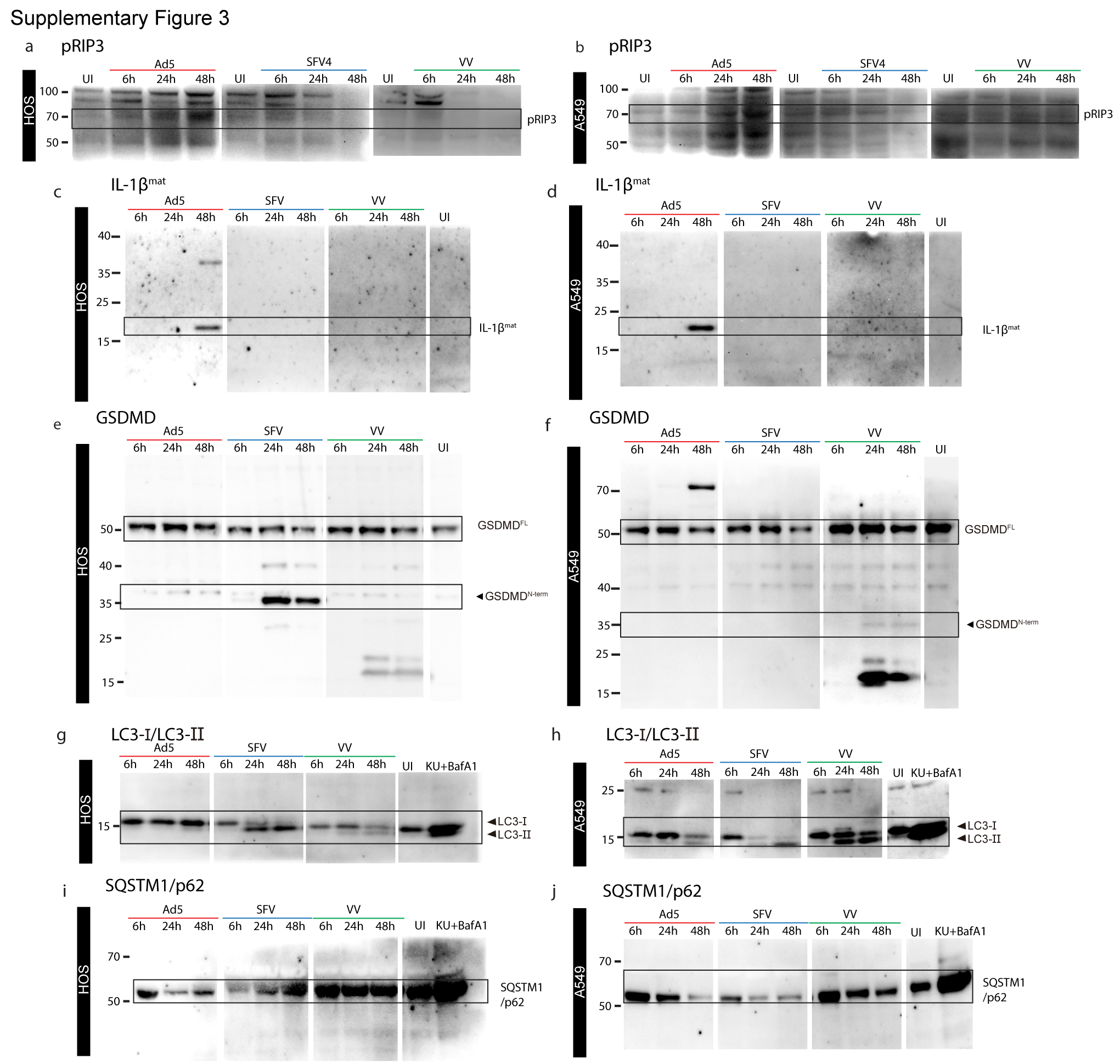

Supplement: Supplementary file 4 — supplementary figure 3 [file 41419_2020_2236_MOESM4_ESM.tif]

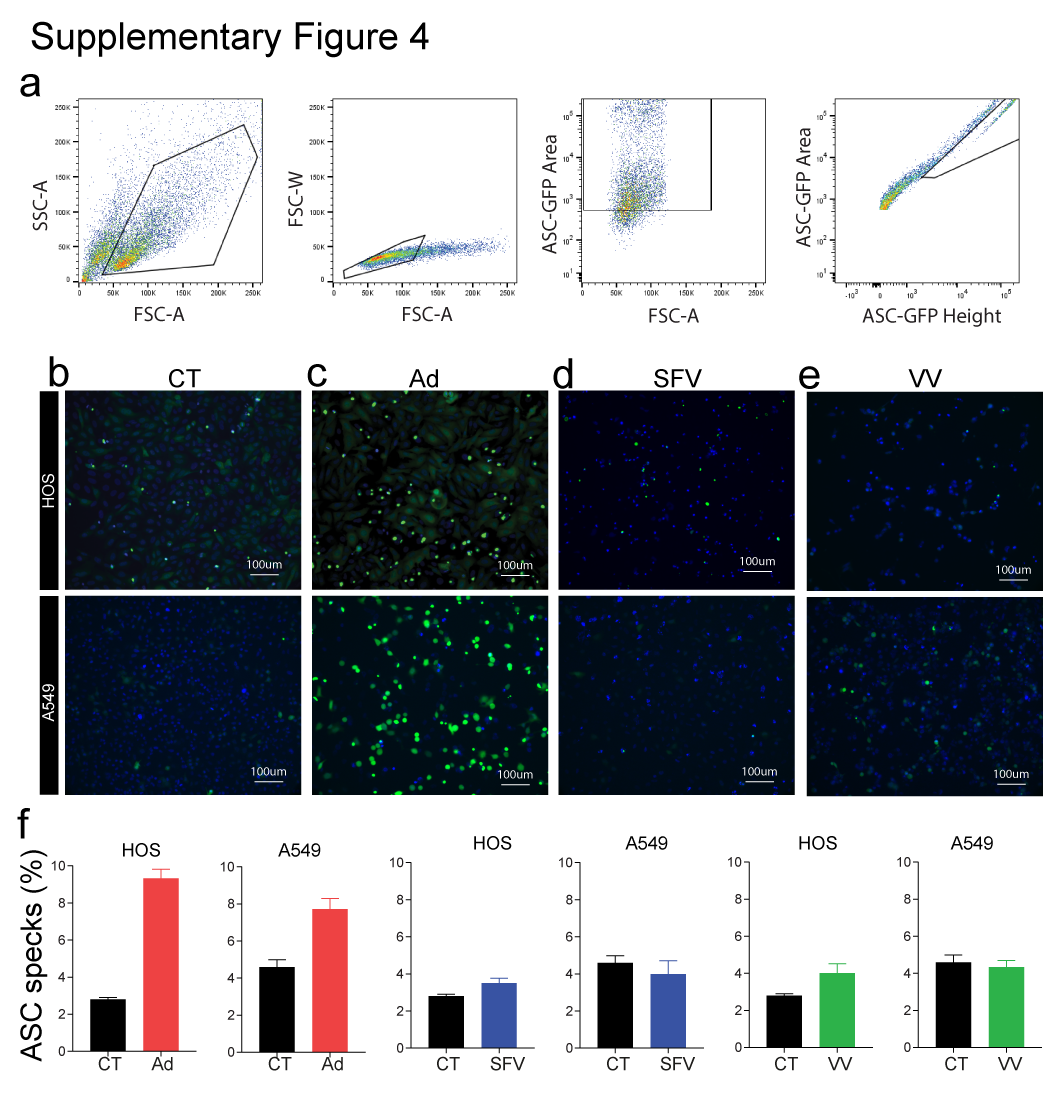

Supplement: Supplementary file 5 — supplementary figure 4 [file 41419_2020_2236_MOESM5_ESM.tif]

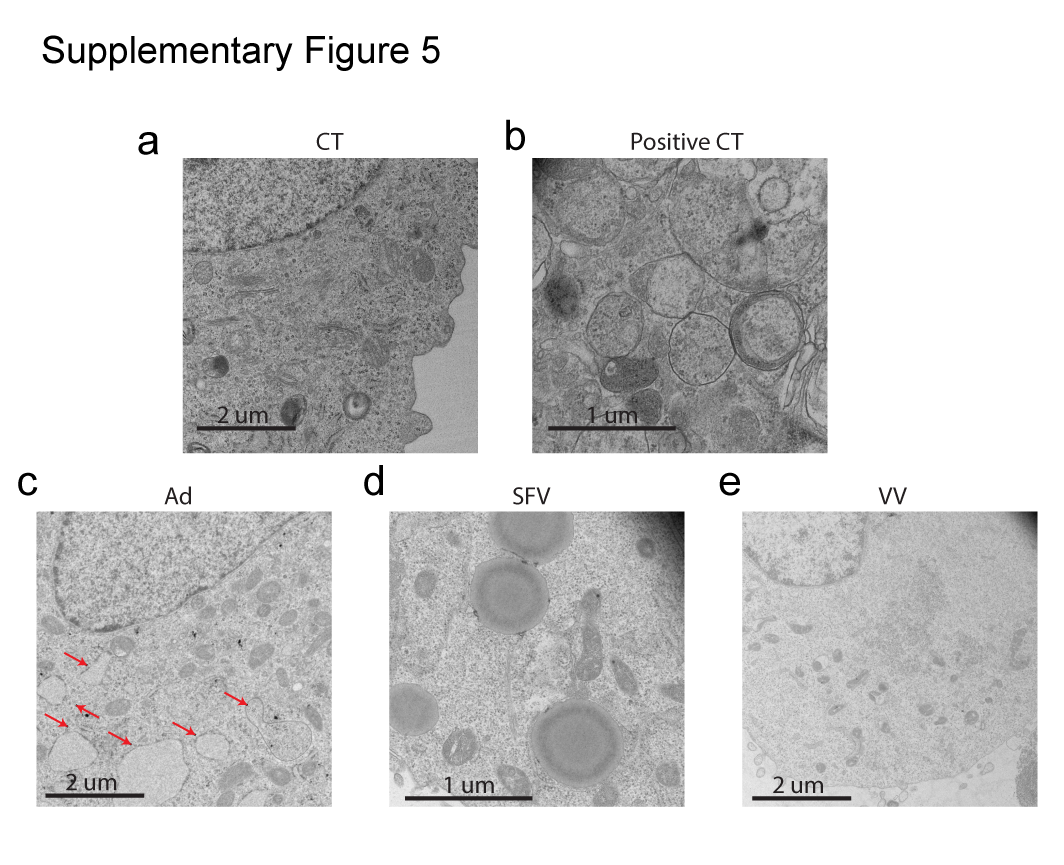

Supplement: Supplementary file 6 — supplementary figure 5 [file 41419_2020_2236_MOESM6_ESM.tif]

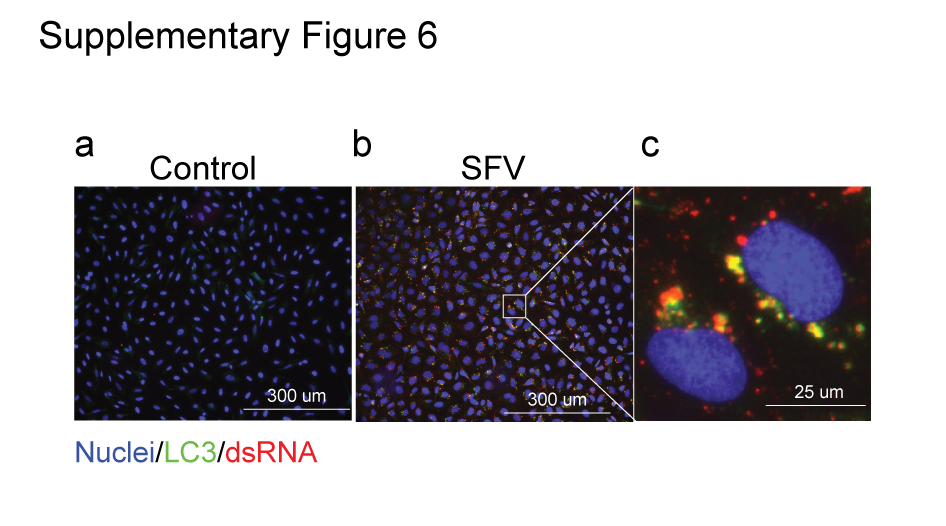

Supplement: Supplementary file 7 — supplementary figure 6 [file 41419_2020_2236_MOESM7_ESM.tif]

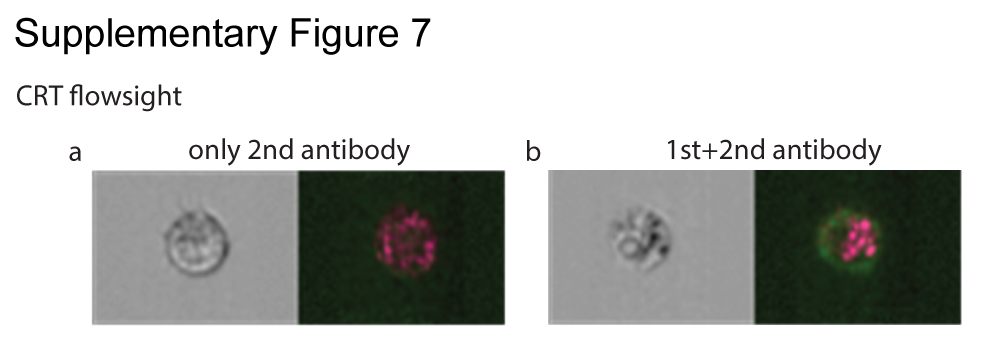

Supplement: Supplementary file 8 — supplementary figure 7 [file 41419_2020_2236_MOESM8_ESM.tif]

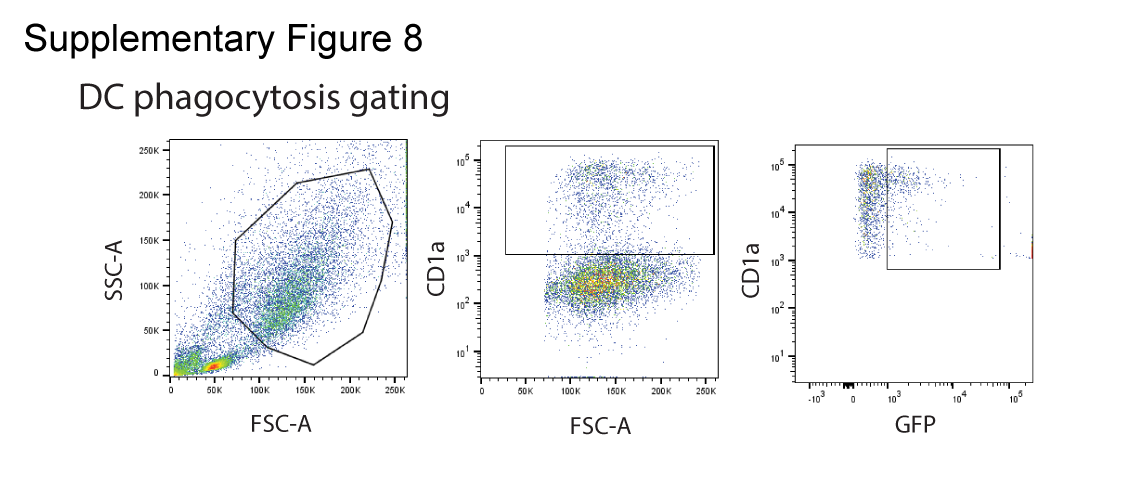

Supplement: Supplementary file 9 — supplementary figure 8 [file 41419_2020_2236_MOESM9_ESM.tif]

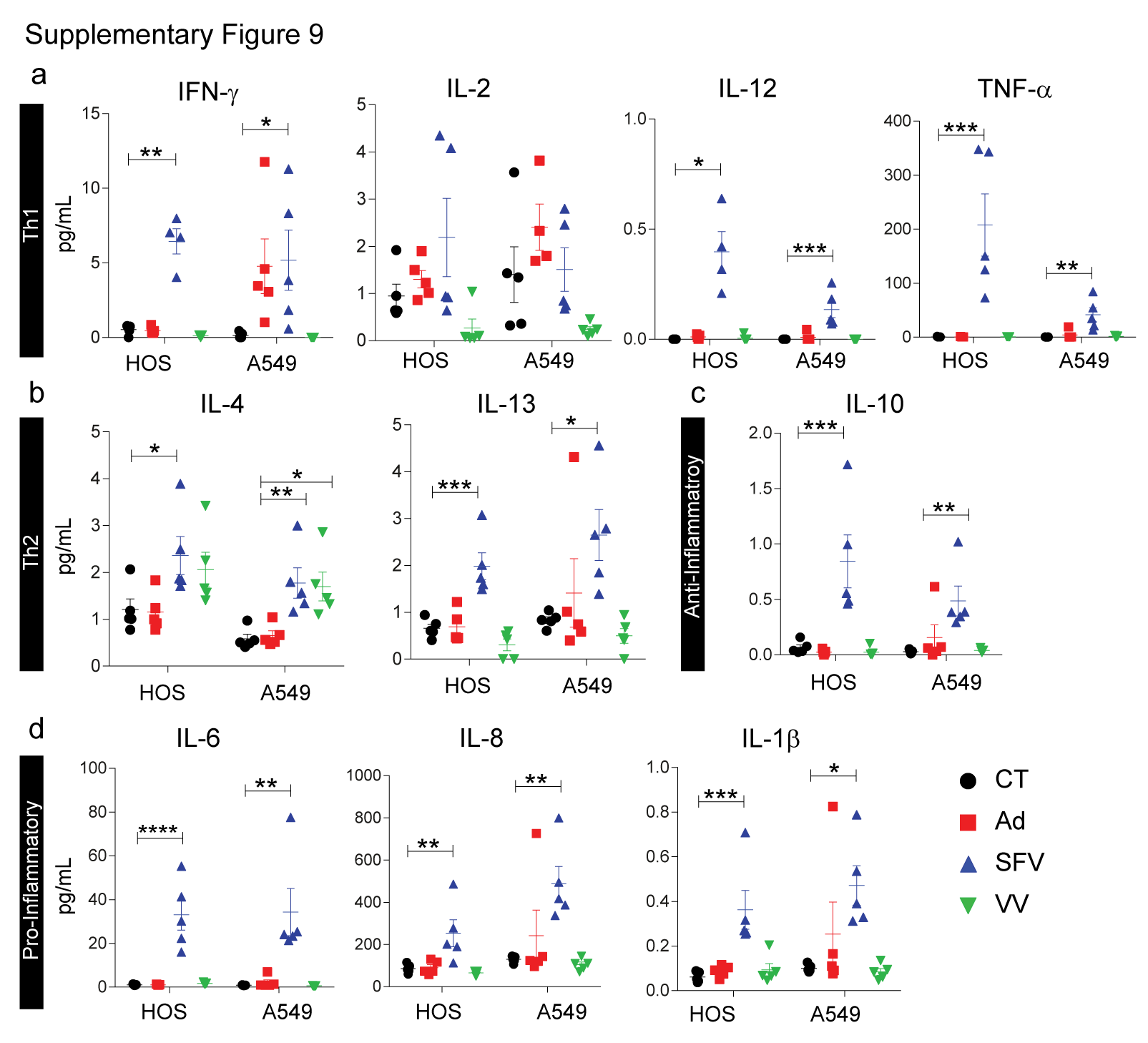

Supplement: Supplementary file 10 — supplementary figure 9 [file 41419_2020_2236_MOESM10_ESM.tif]

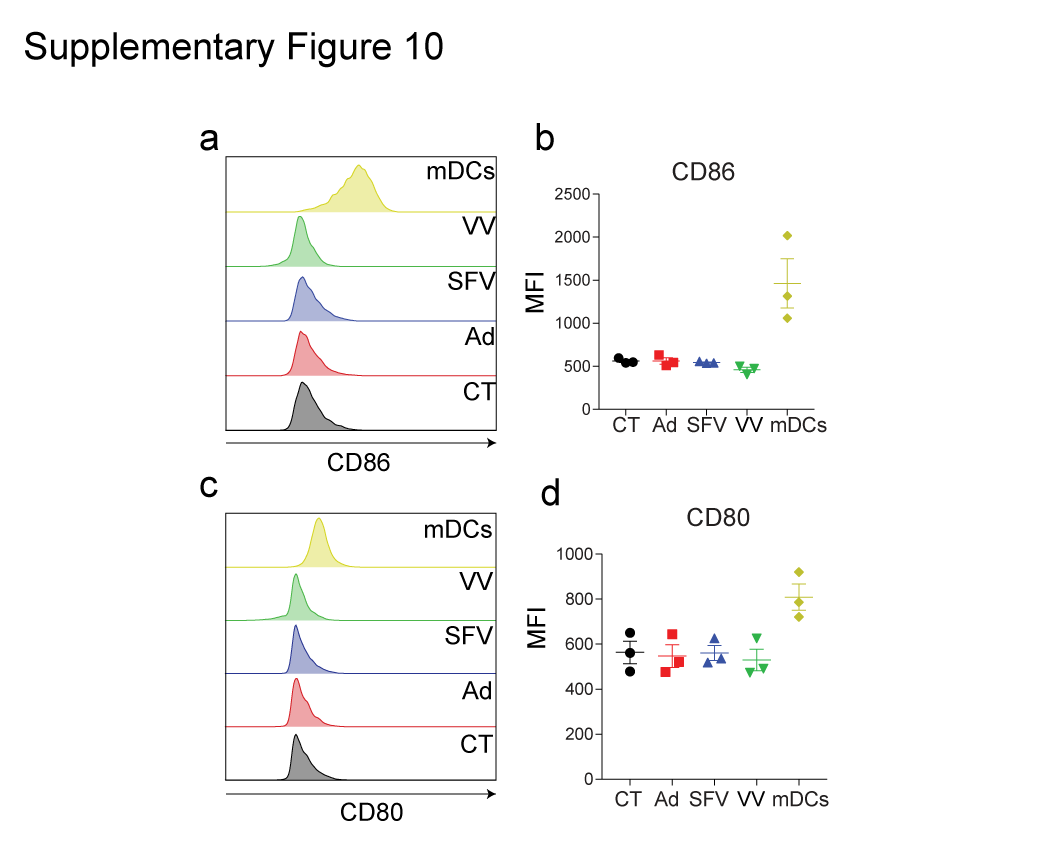

Supplement: Supplementary file 11 — supplementary figure 10 [file 41419_2020_2236_MOESM11_ESM.tif]

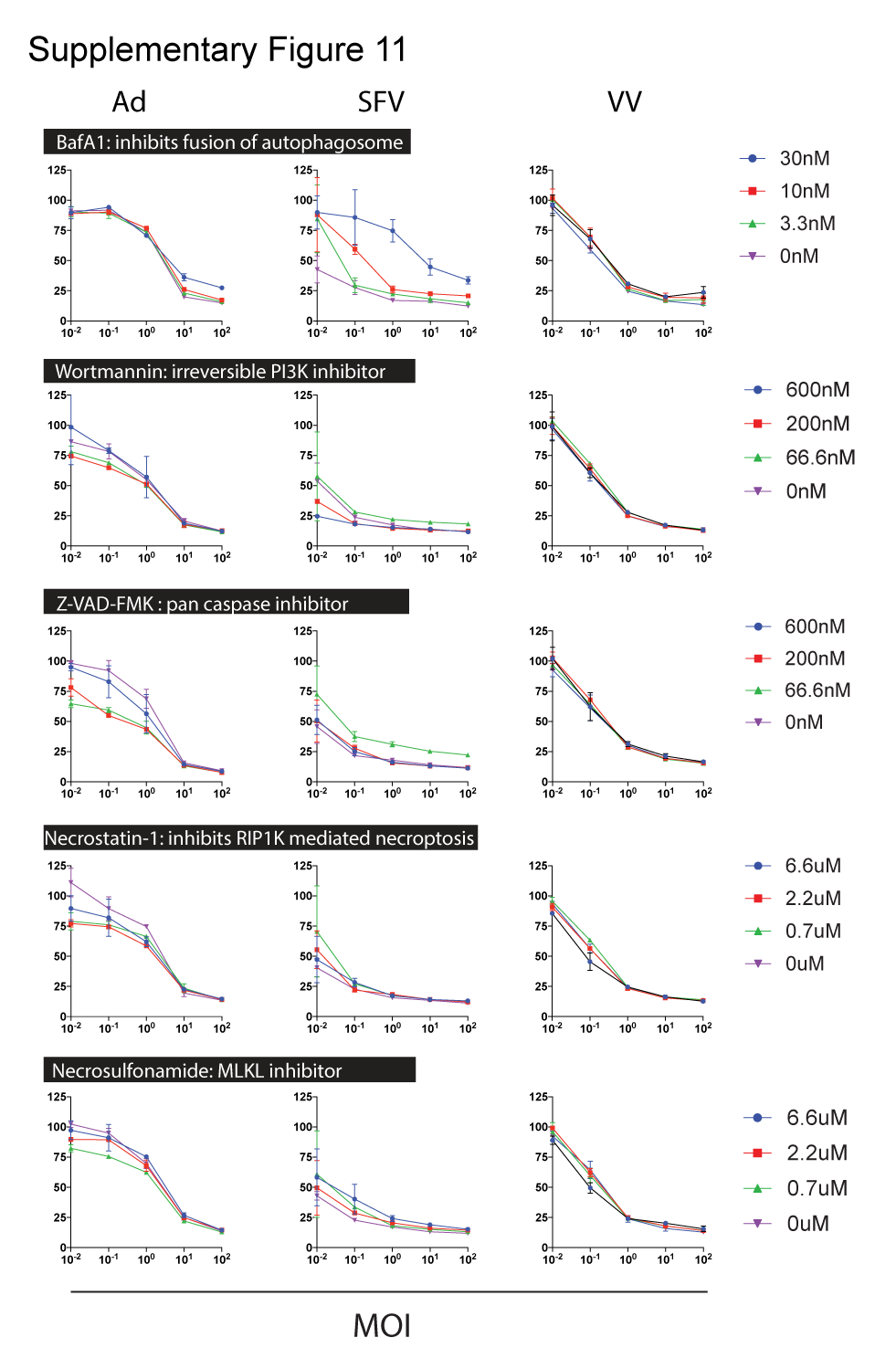

Supplement: Supplementary file 12 — supplementary figure 11 [file 41419_2020_2236_MOESM12_ESM.tif]
